# Data-driven neuroanatomical subtypes of primary progressive aphasia

**DOI:** 10.1093/brain/awae314

**Published:** 2024-10-07

**Authors:** Beatrice Taylor, Martina Bocchetta, Cameron Shand, Emily G Todd, Anthipa Chokesuwattanaskul, Sebastian J Crutch, Jason D Warren, Jonathan D Rohrer, Chris J D Hardy, Neil P Oxtoby

**Affiliations:** Centre for Medical Image Computing, Department of Computer Science, University College London, London WC1V 6LJ, UK; Dementia Research Centre, UCL Queen Square Institute of Neurology, University College London, London WC1N 3AR, UK; Centre for Medical Image Computing, Department of Computer Science, University College London, London WC1V 6LJ, UK; Dementia Research Centre, UCL Queen Square Institute of Neurology, University College London, London WC1N 3AR, UK; Dementia Research Centre, UCL Queen Square Institute of Neurology, University College London, London WC1N 3AR, UK; Dementia Research Centre, UCL Queen Square Institute of Neurology, University College London, London WC1N 3AR, UK; Dementia Research Centre, UCL Queen Square Institute of Neurology, University College London, London WC1N 3AR, UK; Dementia Research Centre, UCL Queen Square Institute of Neurology, University College London, London WC1N 3AR, UK; Dementia Research Centre, UCL Queen Square Institute of Neurology, University College London, London WC1N 3AR, UK; Centre for Medical Image Computing, Department of Computer Science, University College London, London WC1V 6LJ, UK

**Keywords:** subtype and stage inference, progression modelling, phenotype, machine learning, atypical dementia, longitudinal

## Abstract

The primary progressive aphasias are rare, language-led dementias, with three main variants: semantic, non-fluent/agrammatic and logopenic. Although the semantic variant has a clear neuroanatomical profile, the non-fluent/agrammatic and logopenic variants are difficult to discriminate from neuroimaging. Previous phenotype-driven studies have characterized neuroanatomical profiles of each variant on MRI. In this work, we used a machine learning algorithm known as SuStaIn to discover data-driven neuroanatomical ‘subtype’ progression profiles and performed an in-depth subtype–phenotype analysis to characterize the heterogeneity of primary progressive aphasia.

Our study included 270 participants with primary progressive aphasia seen for research in the UCL Queen Square Institute of Neurology Dementia Research Centre, with follow-up scans available for 137 participants. This dataset included individuals diagnosed with all three main variants (semantic, *n* = 94; non-fluent/agrammatic, *n* = 109; logopenic, *n* = 51) and individuals with unspecified primary progressive aphasia (*n* = 16). A dataset of 66 patients (semantic, *n* = 37; non-fluent/agrammatic, *n* = 29) from the ARTFL LEFFTDS Longitudinal Frontotemporal Lobar Degeneration (ALLFTD) Research Study was used to validate our results. MRI scans were segmented, and SuStaIn was used on 19 regions of interest to identify neuroanatomical profiles independent of the diagnosis. We assessed the assignment of subtypes and stages, in addition to their longitudinal consistency.

We discovered four neuroanatomical subtypes of primary progressive aphasia, labelled S1 (left temporal), S2 (insula), S3 (temporoparietal) and S4 (frontoparietal), exhibiting robustness to statistical scrutiny. S1 was correlated strongly with the semantic variant, whereas S2, S3 and S4 showed mixed associations with the logopenic and non-fluent/agrammatic variants. Notably, S3 displayed a neuroanatomical signature akin to a logopenic-only signature, yet a significant proportion of logopenic cases were allocated to S2. The non-fluent/agrammatic variant demonstrated diverse associations with S2, S3 and S4. No clear relationship emerged between any of the neuroanatomical subtypes and the unspecified cases. At first follow-up, subtype assignment was stable for 84% of patients, and stage assignment was stable for 91.9% of patients. We partially validated our findings in the ALLFTD dataset, finding comparable qualitative patterns.

Our study, leveraging machine learning on a large primary progressive aphasia dataset, delineated four distinct neuroanatomical patterns. Our findings suggest that separable spatiotemporal neuroanatomical phenotypes do exist within the primary progressive aphasia spectrum, but that these are noisy, particularly for the non-fluent/agrammatic non-fluent/agrammatic and logopenic variants. Furthermore, these phenotypes do not always conform to standard formulations of clinico-anatomical correlation. Understanding the multifaceted profiles of the disease, encompassing neuroanatomical, molecular, clinical and cognitive dimensions, has potential implications for clinical decision support.

## Teaser

Taylor *et al.* used a machine learning algorithm to identify data-driven profiles of brain atrophy in primary progressive aphasia, from a large dataset of patients' MRI scans. They uncovered four data-driven subtypes, and examined the correspondence between these subtypes and clinical phenotypes.

## Introduction

Primary progressive aphasia (PPA) is a term used to refer to a set of rare, language-led dementias. There are three canonical variants of PPA recognized by consensus diagnostic criteria: semantic (svPPA), non-fluent/agrammatic (nfvPPA) and logopenic (lvPPA).^1^ The classification of PPA into one of the three major variants can occur at one of three levels: clinical, imaging supported or definite pathological. At the clinical level, svPPA is associated with impaired naming and single-word comprehension as the initial manifestations of a pan-modal semantic memory impairment.^1^ The clinical spectrum of nfvPPA is the most diverse of the three canonical variants and is typically associated with agrammatism and/or apraxia of speech.^1-3^ The most recently defined of the PPA variants is lvPPA, having been described first in 2004,^4^ and enshrined in consensus criteria in 2011.^5^ It is typified by impaired word retrieval and impaired repetition of phrases.^1,6^ The term PPA also encompasses further ‘fragmentary’ syndromes,^1,6^ including mixed or atypical presentations.^7-9^ Individuals who do not fit the profile of one of the three main variants but who satisfy overarching criteria for PPA (i.e. a progressive, speech/language-led dementia) can be classified as PPA not otherwise specified (PPA-nos).^8^ As the disorder progresses, people with all PPA variants can experience non-language cognitive impairments, including memory difficulties, executive function deficits and behavioural changes.^10,11^

Neuroimaging can be used to corroborate a diagnosis based on symptom presentation.^1,12,13^ The clearest anatomical progression occurs in svPPA, which is typically associated with left anterior temporal lobe atrophy. Recent studies have identified the initial phase of atrophy in svPPA as beginning in the bilateral amygdala and progressing through the left, then right temporal pole.^14,15^ Neuroimaging findings in nfvPPA, lvPPA and PPA-nos are more variable,^6^ with neuroanatomical similarities between nfvPPA and lvPPA resulting in difficulties in discriminating these phenotypes based on MRI scans alone.^2,16^ A recent study found an association between a clinical diagnosis of nfvPPA and initial atrophy of the left striatum, followed by the bilateral anterior insula, then the left thalamus, with relative sparing of the left precuneus and cuneus.^15^ A study of the neuroanatomical profile of lvPPA identified initial atrophy of the left posterior temporal lobe, with longitudinal involvement of the parietal lobe.^17^ Whether PPA-nos cases share a common underlying neuroanatomical profile is unknown.^9^ There is therefore considerable clinical and research interest in defining the neuroanatomical underpinnings of PPA.

A clinical diagnosis of PPA is typically associated with a primary pathology of frontotemporal lobar degeneration (FTLD) or Alzheimer's disease.^18,19^ There is pathological variation by clinical variant: >80% of svPPA diagnoses are found to be caused by the TAR DNA-binding protein 43 (FTLD-TDP43) proteinopathy at post-mortem^20^; >80% of nfvPPA diagnoses are associated with a primary tauopathy (FTLD-tau)^20^; and >80% of lvPPA diagnoses are associated with Alzheimer's disease pathology.^21^ Genetic cases, typically caused by mutations in progranulin (*GRN*) or more rarely chromosome 9 open reading frame 72 (*C9orf72*), represent a substantial minority of all PPA cases. Genetic PPA has been reported as most predominant in nfvPPA and PPA-nos.^22^

The Subtype and Stage Inference (SuStaIn) algorithm combines disease progression modelling with unsupervised machine learning to identify subgroups of patients based on clustering into similar patterns of atrophy.^23^ Subsequently, the trained model can be applied to cluster patients based on trajectories of neuroanatomy, irrespective of diagnosis or clinical characteristics. SuStaIn has previously been applied in genetic frontotemporal dementia,^24^ Alzheimer's disease^23,25^ and other neurodegenerative diseases.^26^

The overlap of clinical phenotype and neuroanatomical changes in PPA is poorly understood. Previous studies have investigated neuroanatomy within PPA phenotypes,^15,27^ which we sought to advance by providing a probabilistic characterization of neuroanatomy within clinical phenotype and a data-driven discovery of neuroanatomical subtypes of PPA. We applied the SuStaIn algorithm^23^ to a large retrospective dataset of MRI scans from individuals diagnosed with PPA. We analysed model stability in a longitudinal subset of participants with PPA and validated the neuroanatomical subtypes using an external dataset. Finally, we investigated the correspondence between model subtypes and clinical phenotypes to gain a better understanding of the neuroanatomical heterogeneity amongst the canonical variants of PPA. These advancements provide a quantitative template for understanding disease progression and for neuroanatomical assessment/staging of patients, potentially before symptoms appear; they also contribute to our understanding of biological overlap between clinical variants.

## Materials and methods

We performed discovery on a historical dataset from the UCL Queen Square Institute of Neurology Dementia Research Centre in London, UK, and used data from the ARTFL LEFFTDS Longitudinal Frontotemporal Lobar Degeneration (ALLFTD) Research Study, a North American study, as an external test set.

### Sample characteristics

Data were collected from participants enrolled in five longitudinal studies at the Dementia Research Centre, UCL, between 1993 and 2020 (Supplementary Fig. 1). All participants gave informed consent, and ethical approval was granted by The National Hospital for Neurology and Neurosurgery & UCL Institute of Neurology Joint Research Ethics Committee. The Queen Square discovery dataset was compiled retrospectively from these studies, with inclusion criteria of a PPA diagnosis according to clinical consensus criteria^1^ and an MRI scan. The dataset included 270 patients with a diagnosis of PPA (svPPA, *n* = 94; nfvPPA, *n* = 109; lvPPA, *n* = 51; PPA-nos, *n* = 16). Of those with svPPA, *n* = 89 were ‘left predominant’, classified according to whether the left or right temporal pole was more atrophied at baseline.^28,29^ The 53% of cases that were diagnosed prior to 2011 (before the publication of the current diagnostic criteria) were assessed retrospectively by a senior neurologist (J.D.R.) and rediagnosed/relabelled where appropriate. A minority of patients received a secondary clinical diagnosis of an associated neurological condition: Parkinson's disease (PD; *n* = 1), progressive supranuclear palsy (PSP; *n* = 8), corticobasal syndrome (CBS; *n* = 8), PSP/CBS (*n* = 1) and motor neuron disease (*n* = 2).

Histopathological data were available for 51 (18%) patients in the cohort, whose brains were donated to the Queen Square Brain Bank archives, where tissues are stored under a licence from the Human Tissue authority (No. 12198). Both the brain donation programme and protocols have received ethical approval for donation and research by the National Research Ethics Service (NRES) Committee London—Central. Among these cases, 14 presented with Alzheimer's disease pathology, including one case with concomitant Parkinson's disease (AD-PD). Fourteen cases exhibited FTLD-tau pathology, with eight diagnosed as Pick's disease (Tau-Pick's), four as corticobasal degeneration (Tau-CBD), two as globular glial tauopathy (Tau-GGT), and two as progressive supranuclear palsy (Tau-PSP). Additionally, 21 cases presented with FTLD-TDP-43 pathology, with one categorized as type A and 20 as type C.

A range of neuropsychological measures recorded within 3 months of their baseline MRI scan were available for a subset of the patients. Mini-Mental State Examination (MMSE) scores were available for 102 patients, with a mean of 22.3 ± 6.0. A full list of the measures can be found in the Supplementary Material.

Follow-up scans were obtained from 137 (of 270) participants during subsequent research visits. The interval between consecutive scans was 1.1 ± 0.6 years [mean ± standard deviation (SD)]. It is important to note that we included follow-up scans even if the MRI scanner was different from those used at baseline; further details are included in the Supplementary Material.

For the *w*-scoring, we used MRI scans from 121 cognitively normal control subjects, recruited from various studies at the Dementia Research Centre, UCL.

Our test dataset was formed of 66 members of ALLFTD (www.allftd.org/), a large North American natural history study of frontotemporal dementia (FTD), who had a diagnosis of PPA and an MRI scan. This included individuals with svPPA (*n* = 37) and nfvPPA (*n* = 29). There were no individuals with lvPPA in the cohort because one of the inclusion criteria for ALLFTD is assumed frontotemporal lobal degeneration. Neuropsychological test scores were available for a subset of the patients. At least one follow-up scan was obtained for every individual in the dataset. For *w*-scoring of the test dataset, we used MRI scans from cognitively normal control subjects (*n* = 121) recruited from the same ALLFTD study. Table 1 presents the demographic characteristics of the cohort.

**Table 1:** Cohort demographics

Characteristic	PPA	svPPA	nfvPPA	lvPPA	PPA-nos	Control
**Queen Square discovery dataset**						
* n*	270	94	109	51	16	121
Sex	122 F:148 M	40 F:54 M	56 F:53 M	20 F:31 M	6 F:10 M	65 F:56 M
Age at onset, years, mean ± SD	61.8 ± 8.1	59.4 ± 7.6	64.1 ± 8.7	61.5 ± 7.3	60.8 ± 5.7	–
Age at baseline scan, years, mean ± SD	66.1 ± 7.9	64.1 ± 7.1	68.3 ± 8.6	65.8 ± 7.4	64.3 ± 5.7	61.7 ± 11.1
MMSE, (*n*), mean ± SD	(102) 22.3 ± 6.0	(31) 21.3 ± 6.4	(36) 23.4 ± 5.8	(26) 22.0 ± 5.2	(9) 22.4 ± 7.6	–
Total number of visits, mean ± SD	1.9 ± 1.2	2.1 ± 1.4	1.7 ± 0.9	1.9 ± 1.2	2.1 ± 1.6	–
1	133	42	59	24	8	–
2	71	23	30	14	4	–
3	43	17	14	10	2	–
4	23	12	6	3	2	–
Primary pathology, *n*						
Alzheimer's disease	14	0	0	14	0	–
FTLD-tau	16	4	10	0	2	–
FTLD-TDP43	21	19	2	0	0	–
Secondary clinical diagnosis, *n*						
PD	1	0	0	1	0	–
PSP	8	0	8	0	0	–
CBS	8	0	7	0	1	–
PSP/CBS	1	0	1	0	0	–
MND	2	0	2	0	0	–
**ALLFTD test dataset**						
*n*	66	37	29	–	–	121
Sex	34 F:32 M	17 F:20 M	17 F:12 M	–	–	67 F:54 M
Age at baseline scan, years, mean ± SD	64.8 ± 7.1	62.9 ± 6.3	67.2 ± 7.4	–	–	62.3 ± 7.2

Cohort demographics for the full set of neuropsychological measures are available in Supplementary Table 5. ALLFTD = ARTFL LEFFTDS Longitudinal Frontotemporal Lobar Degeneration Research Study; CBS = corticobasal syndrome; F = female; FTLD-tau = frontotemporal lobal degeneration tau; FTLD-TDP43 = frontotemporal lobar degeneration TAR DNA-binding protein 43; lvPPA = logopenic variant PPA; M = male; MMSE = Mini Mental State Examination; MND = motor neuron disease; nfvPPA = non-fluent/agrammatic variant PPA; PD = Parkinson's disease; PPA = primary progressive aphasia; PPA-nos = PPA not otherwise specified; PSP = progressive supranuclear palsy; svPPA = semantic variant PPA.

### Image acquisition and preprocessing

Participants in the Queen Square discovery dataset underwent MRI scans on various 1.5 or 3 T MRI scanners from two different manufacturers (Siemens Trio 3 T, Siemens Prisma 3 T or GE Signa 1.5 T; Supplementary Table 1). Participants in the ALLFTD dataset underwent MRI scans on various 3 T MRI scanners from two different manufacturers (Siemens Trio 3 T or GE Signa 3 T; Supplementary Table 2). In both datasets, cortical and subcortical volumes were parcellated on volumetric T_1_-weighted MRIs using the geodesic information flows (GIF) tool,^30^ which uses the Neuromorphometrics brain parcellation and is based on atlas propagation and label fusion. The total intracranial volume was calculated using SPM12 v.6255, running under MATLAB R2014b (MathWorks, Natick, MA, USA).^31^ All segmentations were inspected visually to ensure accurate segmentation. Only subjects with a usable T_1_-weighted MRI were included. Brain pathology not related to PPA (e.g. brain lesions such as tumours) was an exclusion criterion.

### Statistical analysis

#### Subtype and stage inference algorithm

SuStaIn is an unsupervised machine learning algorithm that identifies disease progression subtypes probabilistically, i.e. it jointly estimates clusters of distinct biomarker trajectories and the trajectories themselves.^23^ These trajectories are characterized as cumulative sequences of biomarkers transitioning from normal to abnormal states, in reference to a control group. In this work, said biomarkers are volumes of segmented brain regions; we refer to the group-level volumetric abnormalities as atrophy. The versatility of SuStaIn has been demonstrated across various neurodegenerative diseases datasets, effectively modelling tau PET heterogeneity in typical Alzheimer's disease,^25^ volumetric abnormalities in typical Alzheimer's disease^23^ and FTD,^24^ and neuroanatomical subtypes in multiple sclerosis.^26^ For our analysis, we used the Python implementation of the SuStaIn algorithm.^32^

At each patient visit, the *w*-score (also known as the covariate-adjusted *z*-score) of brain volume from controls was computed as follows:


(1) $$w\text{-}\mathrm{score}=\frac{\mathrm{observed}\;\mathrm{volume}\;\mathrm{in}\;\mathrm{patient}-\mathrm{predicted}\;\mathrm{patient}\;\mathrm{volume}}{\sqrt{\mathrm{residual}\;\mathrm{variance}}}$$

To calculate the predicted patient volume and residual variance, a linear regression was performed on the controls while accounting for covariates of sex, age at scan, total intracranial volume and scanner types.^14,33^

In the model, biomarker abnormality is determined by reaching user-defined *w*-score thresholds in the respective region. The number of stages in the identified disease trajectories is equal to the total number of abnormality scores across all biomarkers included in the model. This constrains the number of input biomarkers, because in order to prevent overfitting, the total number of stages across all subtypes should not exceed the dataset size.

The selection of regions of interest (ROIs) followed the framework outlined by Scotton *et al*.^34^ In consultation with neurologists and PPA and neuroimaging experts (M.B., J.D.W. and C.J.D.H.), we made a longlist of regions with the greatest abnormality compared with controls, which we narrowed down to a shortlist, which had previously been implicated in PPA^35-37^ and were large enough for reliable segmentation. To streamline the model input and reduce feature complexity, some contiguous subregions were combined into composite regions where this was appropriate anatomically. This resulted in a final list of 19 ROIs (Supplementary Table 3).

For each of the 19 ROIs, we used three *w*-score thresholds (1, 2 and 3), resulting in 57 model events. The choice of using the same integer thresholds across all ROIs was made to aid interpretability; this choice of thresholds is in line with previous applications of SuStaIn.^23,25^ We justified the choice of a maximum *w*-score threshold of three because across all the 19 ROIs, >5% of the patients had reached the most severe score at baseline and thus contained sufficient ‘disease signal’ (Supplementary Fig. 2). The model was fitted on baseline data, with model comparison under 10-fold cross-validation (cross-validation information criterion of up to five subtypes) used to determine the number of subtypes hyperparameter.^23^ Model uncertainty was estimated using 10 000 Markov chain Monte Carlo iterations, and we inspected the Markov chain Monte Carlo trace to confirm satisfactory convergence. Given that the model is initialized with an expectation-maximization algorithm, it does not require a burn-in period.^25^

#### Similarity measure for data-driven subtypes

We supplemented our model comparison analysis with assessment of subtype statistical similarity to inform model parsimony. We assessed subtype similarity by comparing posterior event distributions using the Hellinger distance.^38^ For two discrete probability distributions $P=(\;p_{1},\ldots ,\;p_{k})$ and $Q=(q_{1},\ldots ,\;q_{k})$, the Hellinger distance is defined as:


(2) $$H(P,Q)\;=\;\frac{1}{\sqrt{2}}\sqrt{\overset{k}{\underset{i=1}{\sum }}\;{\left(\sqrt{\;p_{i}}-\sqrt{q_{i}}\right)}^{2}}\;$$

The Hellinger distance between two identical distributions is equal to zero, and the maximal Hellinger distance of one occurs when *P* assigns positive probability everywhere *Q* assigns zero probability, or vice versa. We calculated a reference value of $H_{0}=0.89\pm 0.019$ [95% confidence interval (CI): 0.893, 0.895], which represents the statistical similarity of randomized models (for further details, see the Supplementary Material).

#### Patient subtyping and staging

SuStaIn provides a mechanism for patient subtyping and staging. Patients were assigned to a stage by maximizing their stage likelihood across subtypes.^39,40^ Individuals assigned to stage zero were deemed to not be ‘subtypable’ because their regional brain volumes were comparable to those of controls. Those deemed subtypable were assigned to their most probable subtype.^23^

#### Subtype and stage characterization

We used the χ^2^ contingency test to compare proportions, e.g. when exploring subtype–phenotype relationships. We used one-way ANOVA and paired *t*-tests to compare baseline neuropsychological measures across subtypes. We conducted a multilevel linear regression to compare baseline stage assignment to MMSE, with a *z*-test used to test the hypothesis of non-zero regression gradient.

We visualized the subtypes of neuroanatomical trajectory using positional variance diagrams, which show the posterior distribution of events.^40^ Blocks of colour represent the sequence (numbered by the stages on the *x*-axis) in which regional brain volumes (listed on the *y*-axis) become abnormal. Colours correspond to the severity score threshold reached at that stage (*w*-score 1, red; *w*-score 2, magenta; *w*-score 3, blue). Colour density corresponds to model (un)certainty, with dark colours representing high certainty regarding the position, and lighter colours representing low certainty. We visualized the atrophy pattern of each subtype using BrainPainter.^41^

#### Single phenotype staging

The original dataset was divided based on clinical diagnosis, creating three smaller datasets representing single phenotypes: svPPA, nfvPPA and lvPPA. These data were used to analyse correspondence between the data-driven subtypes and the expected sequence of disease progression in the clinical variants. For each single phenotype dataset, we fitted a single *z*-score progression model^23^ by using the pySuStaIn software^32^ with *N*_subtypes = 1 and all other parameters identical.

#### Changing diagnostic criteria consistency

To assess the impact of the 2011 change in diagnostic criteria to include lvPPA as a clinical phenotype,^1,5^ we conducted a separate analysis on the set of individuals who received their scan during or after 2011. The full details of this analysis can be found in the Supplementary Material.

#### Longitudinal model consistency

The trained model was used to assign longitudinal subtype and stage to patients using available follow-up data. To assess the impact of changing scanner type on our results, we conducted a separate longitudinal analysis on the restricted subset of individuals with longitudinal scans acquired using the same MRI machine as at baseline; the full details of this analysis can be found in the Supplementary Material.

#### Alternative *w*-scores consistency

To check that the model results were not determined by the choice of *w*-score thresholds, we conducted separate analyses, re-running the models with different thresholds. We ran an alternative three-threshold model, with thresholds of (2, 4 and 5), and a two-threshold model with thresholds of (1 and 3). The models were fitted on the baseline Queen Square discovery dataset, with model comparison under 10-fold cross-validation. Further details can be found in the Supplementary Material.

#### External consistency: ALLFTD test set

Patients in the ALLFTD cohort were *w*-scored with respect to controls from the same study. Subtypes and stages were assigned to them using the model trained on the Queen Square discovery dataset.

## Results

### Data-driven neuroanatomical subtypes and stages

**Figure 1:**
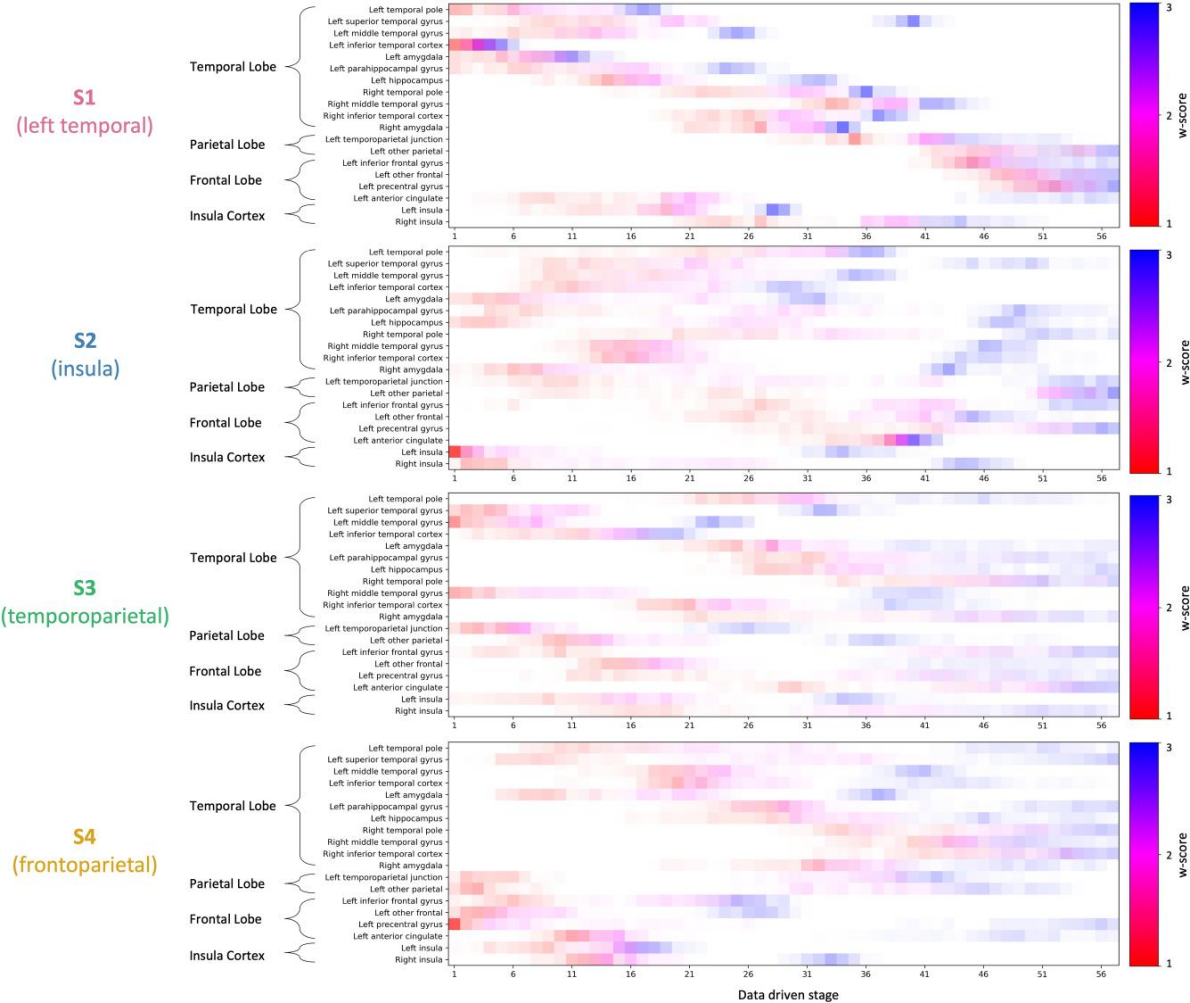
**Positional variance diagrams for the data-driven subtypes.** Along the *y*-axis are the regions of interest used in the model, grouped by location in the brain. The data-driven stages correspond to the sequence in which brain regions become abnormal, with colour representing the degree of abnormality (*w*-score 1, red; *w*-score 2, pink; *w*-score 3, blue) and colour density representing model certainty.

**Figure 2:**
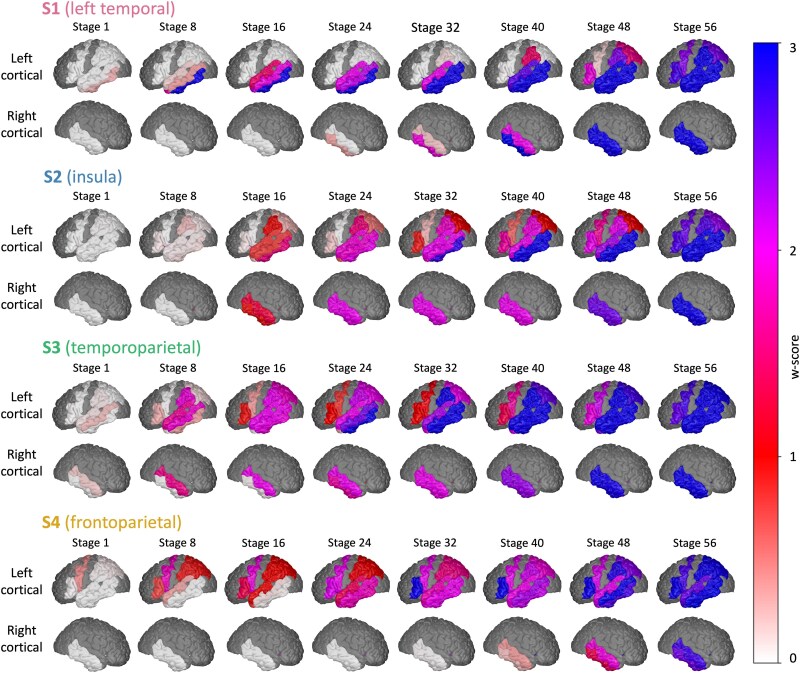
**Stages of regional atrophy in the outer cortical left hemisphere for the data-driven subtypes.** Colour represents the degree of abnormality (*w*-score 1, red; *w*-score 2, pink; *w*-score 3, blue). Brain regions that are dark grey were not included in the model.

Figure 1 shows the four data-driven subtypes of PPA discovered by SuStaIn, supported by cross-validation (Supplementary Fig. 3). Figure 2 shows a visualization of these four subtypes on the brain surface. The identification of four subtypes was supported by statistical pairwise analysis between subtypes, which found low similarity between the positional variance diagrams (Supplementary Table 4).

The first subtype (S1, left temporal) was distinguished by initial atrophy of the left temporal pole, starting in the left inferior temporal cortex, which progressed rapidly through all three *w*-scores. This was followed by atrophy of the insula cortex, then the right temporal pole. There was relative sparing of the left frontal lobe.

In the second subtype (S2, insula) there was early atrophy in the left insula followed by the right insula. This was followed by the left hippocampus, the left parahippocampal gyrus and the amygdala bilaterally. There was relative sparing of the temporal pole bilaterally. The left anterior cingulate was spared until later in the disease, when it progressed through all *w*-scores over <10 model stages, suggesting rapid neurodegeneration.

In the third subtype (S3, temporoparietal) there was initial atrophy in regions around the left temporoparietal junction and the temporal pole. There was relative sparing of the left hippocampus, left amygdala and left parahippocampal gyrus, which did not reach the first *w*-score until a middle stage. Visually compared with S1 and S2, S3 had higher positional variance, owing to either lower sample size or simultaneous events.^42^

The fourth and final subtype (S4, frontoparietal) presented with early atrophy of the left precentral gyrus. This was followed by atrophy of the other frontal lobe regions, then atrophy of the insula cortex. There was relative sparing of the temporal pole bilaterally. Like S3, S4 had higher positional variance.

Across the subtypes, there was a good representation of patients in early and middle stages of the disease, with no-one assigned to a stage above stage 50 of 57 at baseline (Supplementary Fig. 4). In total, six individuals were assigned to data-driven stage zero at baseline and were excluded from all subsequent baseline analysis because they were deemed not ‘subtypable’.

Table 2 records the subtype demographics. Of those who were subtypable, 84% were assigned to a baseline subtype with probability >0.75 (Supplementary Fig. 5). Subtype 1 was the largest subtype, with *n* = 82 (31.0%) patients assigned to it, with an age at first scan of 63.8 ± 6.9 years. Amongst patients assigned to S1, >90% were assigned a disease stage >15, suggesting that most patients assigned to this subtype were beyond the initial disease stages at baseline. Seventy-two (26.9%) patients were assigned S2, with an age at first scan of 66.5 ± 7.2 years; *n* = 59 (22.3%) patients were assigned to S3, with an age at first scan of 66.6 ± 8.3 years; and *n* = 52 (19.7%) patients were assigned to S4, with an age at first scan of 68.4 ± 9.2 years.

**Table 2:** Subtype demographics

Characteristic	S1 (left temporal)	S2 (insula)	S3 (temporoparietal)	S4 (frontoparietal)
**Queen Square discovery dataset**				
* n*	82	71	59	52
Sex	36 F:46 M	34 F:37 M	22 F:37 M	27 F:25 M
Age at onset, years, mean ± SD	59.6 ± 7.5	61.8 ± 7.6	62.1 ± 8.0	64.4 ± 9.0
Age at baseline scan, years, mean ± SD	63.8 ± 6.9	66.5 ± 7.2	66.6 ± 8.3	68.4 ± 9.2
MMSE, (*n*), mean ± SD	(29) 22.5 ± 6.4	(31) 20.8 ± 5.6	(26) 22.2 ± 6.4	(16) 24.9 ± 4.6
Diagnosis, *n*				
svPPA	71	15	4	4
nfvPPA	3	34	21	45
lvPPA	1	18	31	1
PPA-nos	7	4	3	2
Primary pathology, *n*				
Alzheimer's disease	1	2	11	0
FTLD-tau	4	4	1	7
FTLD-TDP43 type C	16	3	1	1
Secondary diagnosis, *n*				
PD	0	0	1	0
PSP	0	2	1	2
CBS	1	4	1	2
PSP/CBS	0	0	1	0
MND	0	1	1	0
**ALLFTD test dataset**				
*n*	23	15	9	14
Sex	11 F:12 M	5 F:10 M	4 F:5 M	9 F:5 M
Age at baseline scan, years, mean ± SD	63.1 ± 6.2	63.2 ± 6.3	68.6 ± 6.4	66.6 ± 8.1
**Diagnosis, *n***				
svPPA	20	10	0	7
nfvPPA	3	5	9	7

Subtype demographics for the full set of neuropsychological measures are available in Supplementary Table 5. ALLFTD = ARTFL LEFFTDS Longitudinal Frontotemporal Lobar Degeneration Research Study; CBS = corticobasal syndrome; F = female; FTLD-tau = frontotemporal lobal degeneration tau; FTLD-TDP43 = frontotemporal lobar degeneration TAR DNA-binding protein 43; lvPPA = logopenic variant PPA; M = male; MMSE = Mini-Mental State Examination; MND = motor neuron disease; nfvPPA = non-fluent/agrammatic variant PPA; PD = Parkinson's disease; PPA = primary progressive aphasia; PPA-nos = PPA not otherwise specified; PSP = progressive supranuclear palsy; svPPA = semantic variant PPA.

### Association with clinical variant and pathology

**Figure 3:**
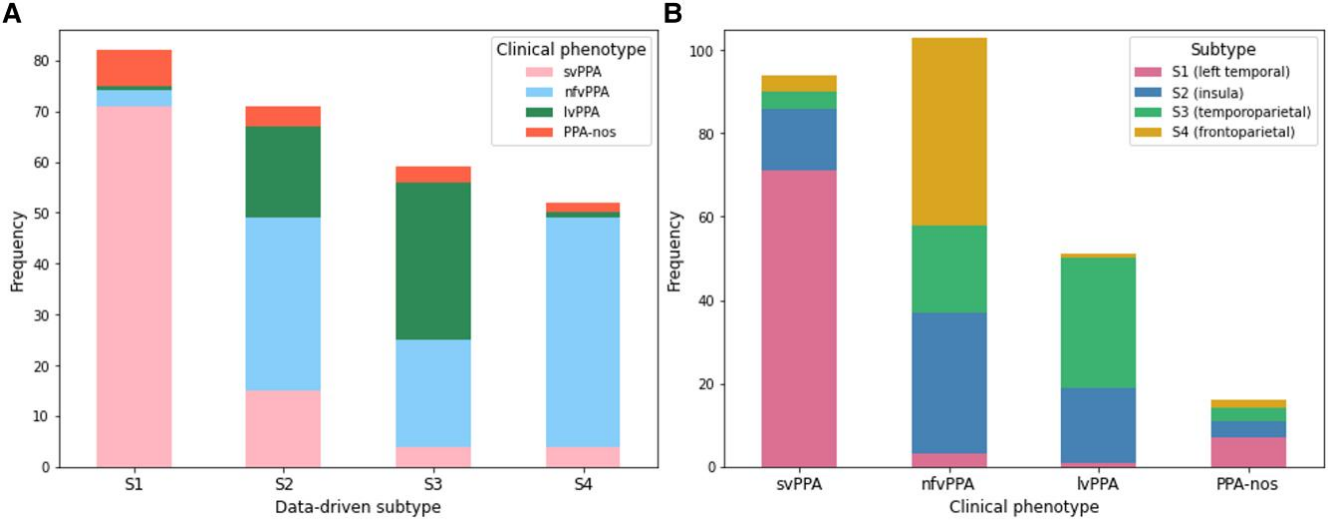
**Comparison between data-driven subtype assignment and clinical diagnosis.** (**A**) The stacked bar chart shows the number of patients with each clinical diagnosis by data-driven subtype assignment. (**B**) The stacked bar chart shows number of patients who were assigned to each data-driven subtype by clinical diagnosis. lvPPA = logopenic variant PPA; nfvPPA = non-fluent/agrammatic variant PPA; PPA-nos = PPA not otherwise specified; svPPA = semantic variant PPA.

Figure 3 shows the association between the clinical diagnosis of each participant and the subtype to which they were assigned. The strongest phenotype–subtype concordance occurred for svPPA, which made up 86.6% of participants in S1 (left temporal), and for nfvPPA, which made up 86.6% of participants assigned to S4 (frontoparietal). The proportion of clinical diagnoses in each subtype was statistically significant [χ^2^(9, *n* = 264) = 269.4, *P* < 0.001].

The strongest pathology–subtype concordance occurred for FTLD-TDP43 type C, for which 16 of 20 (80%) were assigned to S1 (left temporal), and Alzheimer's disease, for which 10 of 13 (76.9%) were assigned to S3 (temporoparietal) (Supplementary Fig. 6).

### Association with neuropsychological test scores

**Figure 4:**
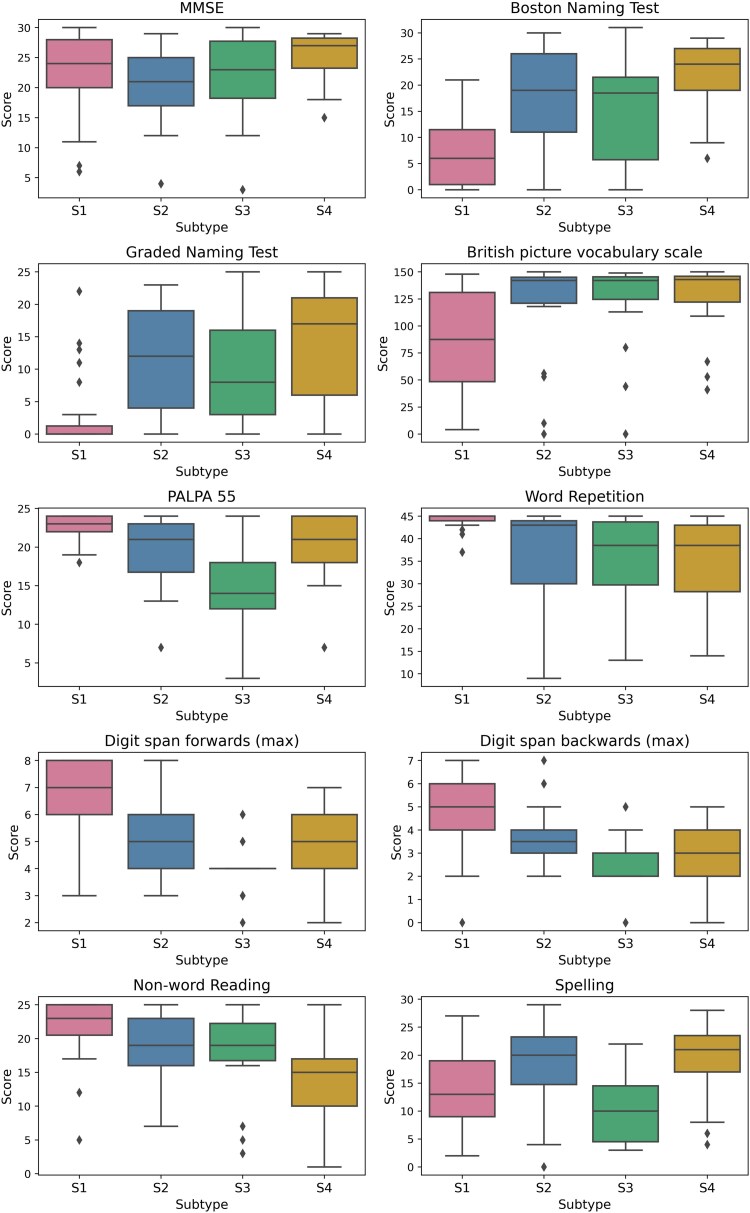
**Boxplots comparing MMSE and neuropsychological scores between subtypes at baseline in the Queen Square discovery dataset.** The full set of neuropsychological scores per subtype is available in Supplementary Table 5. MMSE = Mini-Mental State Examination; PALPA-55 = Psycholinguistic Assessments of Language Processing in Aphasia, subtest 55.

General neuropsychological profiles were in keeping with the syndromic diagnosis for each patient group (Supplementary Table 5), and the S1 subtype was broadly consistent with svPPA. However, neuropsychological profiles of S2, S3 and S4 were more variable and less clearly linked with specific PPA neuropsychological profiles. Figure 4 compares key neuropsychological test scores at baseline, stratified by baseline subtype assignment. A full table, with the count, mean and SD of test scores with reference to normative data, can be found for all available neuropsychological measures in Supplementary Table 5.

Excluding MMSE, all the tests included in Fig. 4 had statistically significant ANOVAs. Excluding MMSE and non-word reading, S1 had statistically different scores in every measure included in Fig. 4 when compared with all other subtypes independently. S1 performed worse on the Boston Naming Test (BNT), the Graded Naming Test (GNT), the British Picture Vocabulary Scale (BPVS) and spelling, but better on the Psycholinguistic Assessments of Language Processing in Aphasia, subtest 55 (PALPA-55; a measure of receptive agrammatism), word repetition and both forwards and backwards digit span. S3 had statistically significant worse scores in both the PALPA-55 and forward digit span when compared with all other subtypes independently. S2 and S4 differed statistically from one another only on the MMSE, in which S4 scored better, and the non-word reading test, in which S4 scored worse.

**Figure 5:**
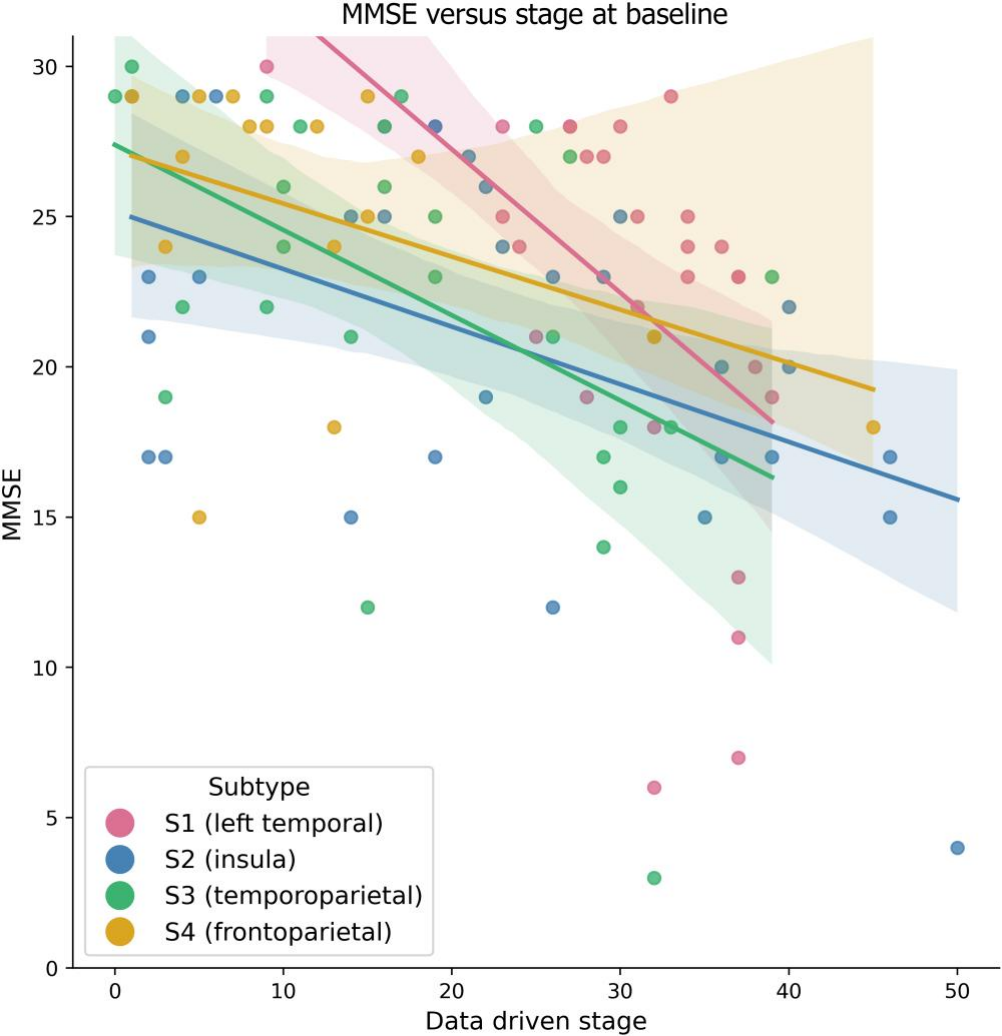
Mini-Mental State Examination (MMSE) versus stage at baseline in the Queen Square discovery dataset.

Figure 5 shows the comparison between baseline data-driven stage and MMSE score, stratified by subtype assignment. Performing a multilevel linear regression, we found that the relationship (regression slope) between MMSE and baseline stage was statistically significant in S1 (*P* = 0.01), S2 (*P* = 0.03) and S3 (*P* = 0.03), but not in S4 (*P* = 0.13).

### Comparison with single phenotype staging

Supplementary Fig. 7 shows the positional variance diagrams for the three clinical phenotypes. A diagnosis of svPPA was associated with early decline of the left inferior temporal cortex, followed by decline across the other left temporal regions, then the insula cortex, then the right temporal regions and, finally, decline of the frontal regions. A diagnosis of nfvPPA was associated with initial decline of the left precentral gyrus, then the other left frontal lobe regions and the parietal lobe, then the insula cortex, closely followed by the temporal lobe. A diagnosis of lvPPA was associated with initial decline of the left middle temporal gyrus, followed by the superior temporal gyrus, left inferior temporal cortex and left temporoparietal junction, with late sparing of the right temporal pole and the left anterior cingulate.

There was a statistically significant correlation between the neuroanatomical progression in S1 (left temporal) and the svPPA phenotypic cohort (Hellinger distance, 0.44), and between S3 (temporoparietal) and the neuroanatomical progression in the lvPPA phenotypic cohort (Hellinger distance, 0.46) (Supplementary Table 6).

### Longitudinal data-driven subtypes and staging

**Figure 6:**
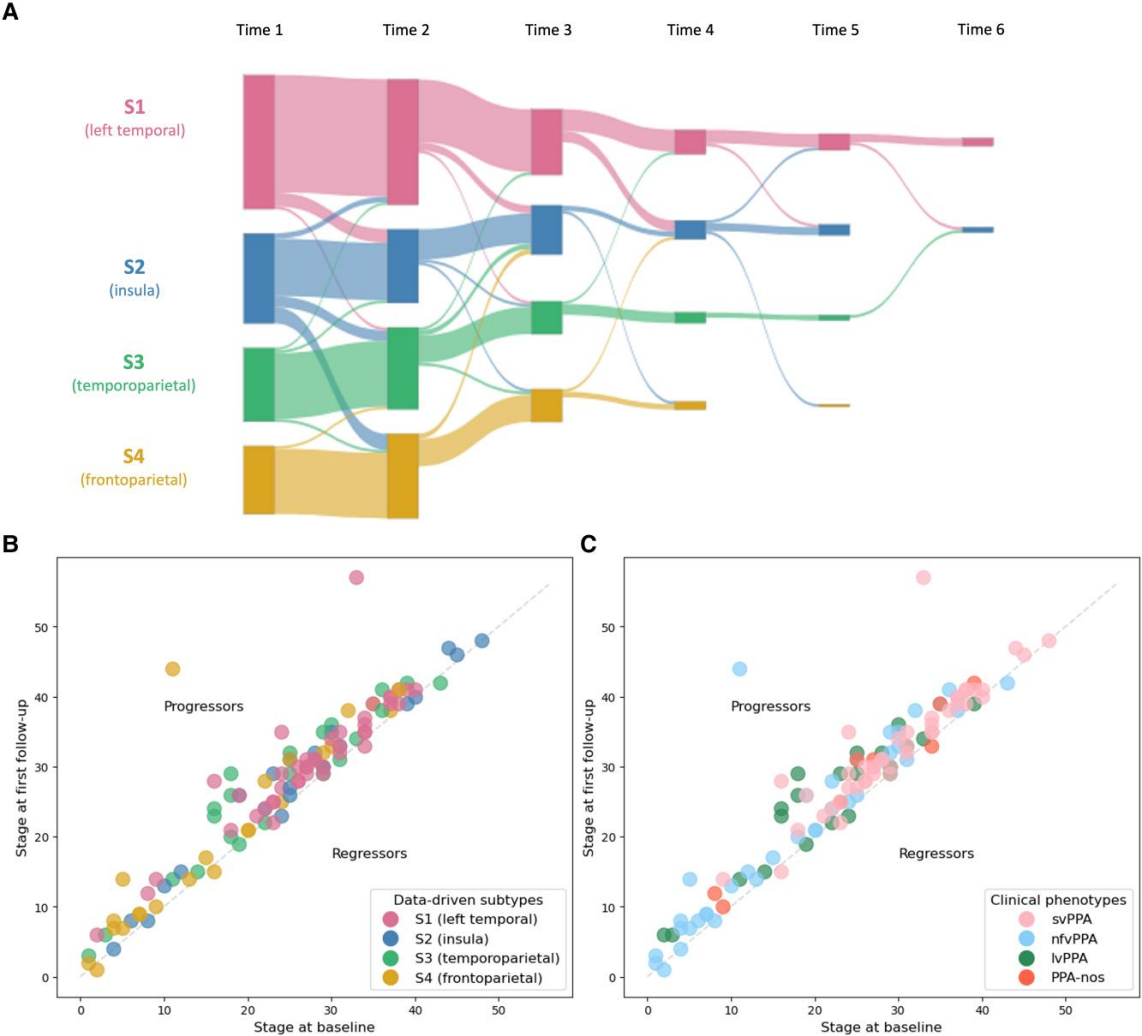
**Longitudinal consistency of subtype and stage assignment.** (**A**) Sankey diagram of subtype assignment between baseline and first follow-up visits. (**B**) Data-driven stage assignment at baseline and first follow-up by data-driven subtype. (**C**) Data-driven stage assignment at baseline and first follow-up by clinical subtype. Those above the diagonal progressed at follow-up, whereas those below regressed. lvPPA = logopenic variant PPA; nfvPPA = non-fluent/agrammatic variant PPA; PPA-nos = PPA not otherwise specified; svPPA = semantic variant PPA.

Figure 6A is a Sankey diagram demonstrating the longitudinal assignment to subtype for the 135 of 137 patients who had longitudinal data and were subtypable at baseline. Subtype consistency between first and second MRI scan was 87.8%/63.6%/88.9%/96% for S1/S2/S3/S4, respectively. Of the 112 of 134 (84%) patients whose subtype assignment was stable at the first two time points, the mean probability of baseline subtype assignment was 0.93.

In addition to having the most variability in longitudinal subtype assignment (63.6%), S2 also had the lowest probability of subtype assignment at baseline. This reflects the fact that those assigned to S2 at baseline who then changed subtype were assigned to the subtype with a lower baseline probability and with earlier stage assignment than those whose assignment of S2 was stable (Supplementary Fig. 1).

Figure 6B and C shows the longitudinal consistency of model staging in the 112 of 134 individuals with stable subtype assignment across the first two time points, stratified by model subtype (Fig. 6B) and clinical diagnosis (Fig. 6C). At first follow-up, 96 of 112 (85.7%) patients advanced (upwards) to a later stage, 10 of 112 (8.9%) patients remained at the same stage (on the diagonal), and a further 6 of 112 (5.4%) patients regressed (downwards) to an earlier stage, giving a longitudinal staging consistency of >94.6% (106 of 112).

Of the 66 patients having three or more MRI scans and subtypable at baseline, 39 were assigned to the same subtype at every time point. Amongst these 66 patients, 49 were assigned to monotonically increasing stages across the time points.

### Internal consistency

The four data-driven subtypes were consistent across various subsets of the data and alternative model hyperparameters. All the models using alternative *w*-score thresholds replicated the four subtypes found in the primary analysis. The analysis of the subset of individuals who were scanned after 2010, hence after lvPPA was enshrined in the consensus criteria, was consistent with the findings in the main Queen Square discovery dataset. The analysis of the subset of patients scanned on the same MRI scanner type longitudinally was consistent with the longitudinal analysis of the full dataset Queen Square discovery dataset.

### Results of ALLFTD test dataset

In the test set, five patients were assigned to data-driven stage zero, hence excluded from subsequent analysis. Of those who were subtypable, 23 patients were assigned to S1, with an age at first scan of 63.1 ± 6.2 years, 15 patients were assigned to S2, with an age at first scan of 63.2 ± 6.28 years, nine patients were assigned to S3, with an age at first scan of 68.6 ± 6.4 years, and 14 patients were assigned to S4, with an age at first scan of 66.6 ± 8.1 years.

The findings in the ALLFTD dataset replicated the Queen Square discovery dataset. As in the Queen Square discovery dataset, the greatest phenotype subtype concordance was between svPPA and S1 (left temporal) (54.1% of svPPA were assigned to S1). Subtypes and stages were stable at follow-up clinic visits, with >90% of individuals remaining in the same stage or advancing to a later stage at first follow-up. Full details of the results of the ALLFTD test dataset can be found in the Supplementary Material.

## Discussion

In this study, we aimed to understand neuroanatomical disease progression in PPA, and specifically, how neuroanatomical subtypes relate to clinical phenotypes. Our retrospective cohort was one of the largest PPA datasets compiled to date and included individuals with the three named clinical variants and with PPA-nos. Our model identified four statistically different trajectories of PPA disease progression based on volumetric markers extracted from MRI scans. Secondary analysis compared these data-driven subtypes with the atrophy patterns of the clinical phenotypes, revealing subtle differences between the two. Reassuringly, the patterns of change in brain volume in the clinical phenotypes identified in this analysis were consistent with previous findings.^1,35^ The application of SuStaIn across analyses facilitated comparison between neuroanatomical profiles, ultimately contributing a unique data-driven perspective on relationships between clinical phenotype and spatiotemporal neuroanatomical pathology subtypes in PPA. We do not aim for the data-driven subtypes identified in this work to replace clinical definitions of the disease. Rather, this work serves as a step towards a biological definition of PPA, something that has already occurred in Alzheimer's disease^43^ and is beginning to happen in Parkinson's disease.^44,45^

There was a strong correspondence between S1 and svPPA, with the disease progression profile of S1 closely matching the reported patterns of initial temporal pole atrophy in svPPA,^35,46^ evolving to implicate the contralateral hemisphere later in the disease.^47^ In particular, the sequence of decline in S1 overlapped with stages of atrophy reported in TDP-43 type C, comprising left temporal pole, then the left insula, then the insula.^14^ Analysis of neuropsychological test scores identified impaired object naming and single word comprehension in S1, with preserved speech output and grammar in line with svPPA.^1,2^ Individuals assigned to S1 were largely assigned to stages midway through the disease progression, even at baseline. This reflects the fact that diagnosis typically occurs later for individuals with PPA compared with typical Alzheimer's disease,^48,49^ particularly in svPPA,^10,11^ hence exhibiting relatively advanced accumulation of brain differences at baseline. A side effect is that analyses of atrophy in svPPA struggle to distinguish the very earliest regions of change,^14,50^ as seen in S1, in which initial progression through the left temporal pole regions was all staged together. Despite this, S1 was the clearest and most consistent subtype across analyses, matching the notion that svPPA is the most coherent PPA variant clinically, pathologically and neuroanatomically.^2^

Phenotype–subtype correspondence of the other subtypes was more complicated. The most pronounced correspondence was between S3 and lvPPA; S3 had a high statistical similarity to the disease progression seen in the single phenotype lvPPA model, and 60.8% of patients with an lvPPA diagnosis were assigned to it. Of the data-driven subtypes, the sequence of atrophy changes in S3 was more scattered across brain regions, aligning with research showing that atrophy in lvPPA presents as more diffuse.^35^ Furthermore, tests of working memory and object naming were impaired in S3, in line with what is expected in lvPPA.^1,2^ However, S2 was also associated with lvPPA (alongside nfvPPA, which had associations with all subtypes). Recent studies exploring heterogeneity in lvPPA have proposed that both the syndrome itself and the sequence of neuroanatomical changes should be understood in a multidimensional manner,^51,52^ which coincides with the absence of a single lvPPA-specific neuroanatomy amongst the data-driven subtypes in our model.

Results from the Queen Square dataset suggest that S4 could represent a neuroanatomical subtype of nfvPPA, because this subtype was associated with initial left lateralized atrophy of the frontal and temporal lobe regions, which coincides with neuroanatomy reported in a predominantly agrammatic presentation of PPA.^37^ However, as mentioned above, S2 was also associated with nfvPPA. Both S2 and S4 had impaired receptive grammatical processing and speech production, but relatively well-preserved object naming, which is consistent with consensus criteria for nfvPPA.^1,2^ A distinguishing feature of S2 was early insula involvement. Given that the insula is integral to homeostatic and interoceptive function, it would be interesting to look at whether this subtype is associated with early behavioural changes, which have been identified as some of the earliest symptoms in svPPA, nfvPPA and lvPPA.^10,11^

Regarding the association between neuroanatomical subtypes and PPA-nos, no clear pattern emerged. This might be because the label of PPA-nos encompasses heterogeneous disease presentations and potentially diverse neuroanatomical profiles.^8,53^ However, it could also stem from the challenge of identifying data-driven patterns within a relatively small sample size (*n* = 16). The number of individuals diagnosed with PPA-nos in the cohort was low, considering recent findings indicating that as many as 40% of PPA diagnoses might not align with the clinical consensus criteria for any of the three canonical PPA variants.^8,54^ Prospective research in large, well-characterized cohorts of PPA-nos participants will be required to tease apart the neuroanatomical profiles of these cases fully.

Our discoveries, revealing four separable neuroanatomical profiles within the PPA spectrum add to current discussions around the conceptualization of PPA as a spectrum of disorders^37,55^; here, we have shown that separable spatiotemporal neuronatomical phenotypes do exist, but that they are somewhat noisy. Furthermore, these phenotypes do not always conform to standard formulations of clinico-anatomical correlation, suggesting perhaps that these might be determined by specific patterns of pathogenic protein spread over neural networks, for which ‘readouts’ different from traditional neuropsychological tests might be required. This perspective is supported by previous PPA research showing that single neuroanatomical profiles can be linked to multiple clinical phenotypes, whereas canonical phenotypes have been observed to align with diverse neuroanatomical patterns,^13^ as evidenced by ≤62% of individuals with PPA displaying phenotypic attributes that correspond to multiple distinct syndromes.^55^ Moreover, research into the data-driven neuropsychological profiles of PPA has also identified the lack of perfectly aligned phenotypes.^52,53,56^ Ramanan *et al*.^56^ found four principal components of cognition in PPA (general cognition, semantic memory, working memory and motor speech/phonology changes), concluding that the variability in how individuals aligned with these profiles supported the need for a more transdiagnostic approach.

Our results are robust longitudinally and across datasets. Not only did we find that most individuals were assigned a data-driven subtype that was consistent over time, but the findings were also consistent when we excluded individuals whose scanner type varied longitudinally,^1,5^ supporting the inclusion of longitudinal data collected across different scanners. The data-driven model was also consistent across both our in-house discovery dataset and the external test dataset, underscoring the robustness of the identified subtypes. Our analysis was also robust to excluding patients diagnosed prior to 2011, substantiating the inclusion of the pre-2011 lvPPA cases which were rediagnosed.

### Limitations and future work

Here, we have reported on a retrospective analysis of one of the largest PPA datasets compiled to date. However, we note several limitations that suggest opportunities for future research. Model certainty and results would be improved by having larger sample sizes still, which would facilitate investigation into whether the positional variance of events in the disease progression is reflective of genuine variation in neuroanatomy versus an underpowered sample size. The absence of participant education levels or ethnicity details makes it challenging to gauge the representativeness of the sample within the broader population of individuals diagnosed with PPA. Moreover, genetic FTD could account for some of the heterogeneity, because previous research has found that neuroanatomical profiles vary between the hereditary and sporadic variants; for example, there is greater posterior involvement in genetic nfvPPA.^22^ Future work should seek to validate these findings prospectively in large multimodal datasets that are likely to require multicentre, international collaboration.

Further work could leverage clinical knowledge to choose a different subset of ROIs for analysis. We chose a subset of largely cortical ROIs; including more subcortical regions would be interesting, because research has found early involvement of the thalamus and cerebellum in genetic FTD.^57,58^

From the computational perspective, a clustering algorithm might be too blunt a tool to understand the heterogeneity in pathology underpinning the PPA phenotypes, even with a clustering algorithm such as SuStaIn, which takes into account disease accumulation. Future work should seek to develop models that can explicitly model mixtures of subtypes and how disease progression diverges and converges across subtypes. We envisage that such a model would be particularly useful for understanding potential similarities between heterogeneous syndromes, which could provide insights into how disease-modifying treatments could be used across different phenotypes.

## Conclusion

In this study, we identified four distinct patterns of neuroanatomy in clinically diagnosed PPA, with a clear correspondence between S1 and svPPA and with a more variable association between S2/S3/S4 and nfvPPA/lvPPA. Understanding the neuroanatomical, molecular, clinical and cognitive profile of PPA will be crucial for tracking disease progression, screening and stratification for clinical trials, and for developing disease-modifying drug treatments. Although our work benefitted from one of the largest PPA datasets assembled to date, future work should focus on validating these findings in other datasets with additional data modalities, facilitating further comparison of subtype distinctions.

## Supplementary Material

awae314_Supplementary_Data

## Data Availability

The Queen Square dataset that supports the findings of this study is available from the corresponding author, upon reasonable request. The ALLFTD dataset is available upon reasonable request from https://www.allftd.org/data.
