# Supplementary material for: Data-driven neuroanatomical subtypes of primary progressive aphasia
Source: Brain. 2024 Oct 7;148(3):955–68. doi: 10.1093/brain/awae314 (PMC11884653; doi:10.1093/brain/awae314)
Supplement: awae314_Supplementary_Data [file awae314_supplementary_data.zip › brain-2024-01149-File010.pdf]

Supplementary material

Table of Contents

Queen Square discovery dataset *2*
Baseline scans 2
Scanner types 3
Scanner types ALLFTD 3
Regions of interest *4*
Model hyperparameters *5*
w-score thresholds 5
Model fitting 6
Hellinger distance similarity measure for data-driven subtypes 7
Results: Queen Square discovery set *7*
Stage and subtype assignment 8
Association with clinical phenotypes 10
Phenotypic disease progression 10
Phenotypic disease progression vs. Subtype disease progression 13
Longitudinal analysis 15
W-score sensitivity analysis 16
Longitudinal analysis of stable scanner type 20
Post 2010 Dataset *22*
Post 2010 demographics 23
Post 2010 results 23
Two subtype model 24
Four subtype model 26
Post-2010 summary 29
Results: ALLFTD test set *29*
ALLFTD association with clinical phenotypes 29
ALLFTD Longitudinal analysis 30
ALLFTD Association with neuropsychological test scores 31
References *33*

Queen Square discovery dataset

Baseline scans

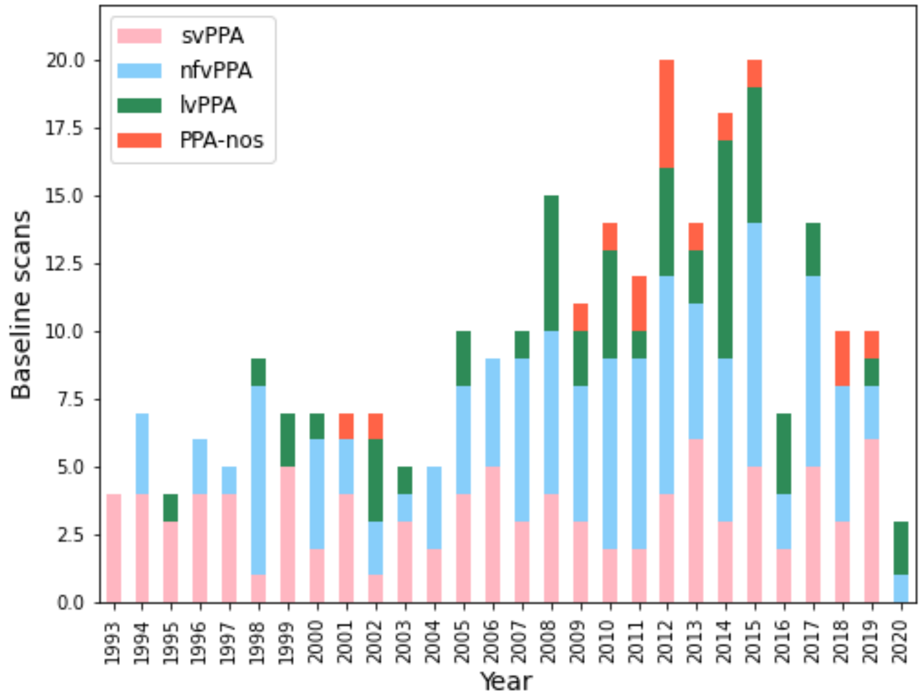

Supplementary Figure 1: **Bar chart of baseline scans by year.**

Abbreviations: svPPA – semantic variant PPA, nfvPPA – nonfluent/agrammatic variant PPA, lvPPA – logopenic variant PPA, PPA-nos – PPA not otherwise specified.

Baseline and follow-up data was collected from participants in five FTD studies at the UCL Queen Square Institute of Neurology Dementia Research Centre between 1993 and 2020. These were: Brain signatures of auditory information processing in the degenerative dementias (06N032, approved by The National Hospital for Neurology and Neurosurgery & UCL Institute of Neurology Joint Research Ethics Committee); Familial and sporadic non-Alzheimer degenerative dementia: longitudinal clinical, biochemical and neuroimaging studies (02/N099, approved by The National Hospital for Neurology and Neurosurgery & UCL Institute of Neurology Joint Research Ethics Committee); Longitudinal Investigation of Fronto-Temporal Dementia and associated disorders (LIFTD) (150805, approved by University College London Hospitals & University College London Joint Research Office); The burden of frontotemporal dementia: clinical course, outcome and caregiving (97/N077,

approved by Joint Medical Ethics Committee, The National Hospital for Neurology & Neurosurgery); Impaired language output in frontotemporal lobar degeneration: a cross-sectional and longitudinal clinical, neuropsychological and neuroimaging study (05/Q0512/99, approved by The National Hospital for Neurology and Neurosurgery & UCL Institute of Neurology Joint Research Ethics Committee).

Scanner types

Supplementary Table 1 details the three different types of MRI scanners, and acquisition parameters used to acquire the images in the Queen Square discovery dataset.

**Type**	**3T Trio**	**3T Prisma**	**1.5T Signa**
Vendor	Siemens, Erlangen, Germany	Siemens, Erlangen, Germany	GE Medical systems, Milwaukee, WI
Scans in study (patients:controls)	114:46	42:27	114:48
Repetition time (ms)	2200	2000	12
Inversion time (ms)	900	850	650
Echo time (ms)	2.9	2.93	5
Acquisition matrix	256x256	256x256	256x256
Spatial resolution (mm)	1.1	1.1	1.5

Supplementary Table 1: Scanners used in data collection of Queen Square discovery dataset.

Scanner types ALLFTD

Supplementary Table 2 details the three different types of MRI scanners, and acquisition parameters used to acquire the images in the ALLFTD test dataset.

**Type**	**3T Trio**	**3T Signa**
Vendor	Siemens, Erlangen, Germany	GE Medical systems, Milwaukee, WI
Scans in study (patients:controls)	65:111	1:6
Repetition time (s)	2.3	2.3
Inversion time (s)	0.9	0.9
Echo time (ms)	2.98	2.98
Acquisition matrix	160x160	166x166

Spatial resolution (mm)	1.2	1.2

Supplementary Table 2: Scanners used in data collection of Queen Square discovery dataset.

Regions of interest

Supplementary Table 3 records the 19 brain regions of interest (ROIs) included in the model.

		ROI	Sub-regions	Queen Square discovery dataset	ALLFTD test dataset
				w-score	w-score
1	Temporal Lobe	Left temporal pole		-2.5 (2.0)	-2.5 (2.2)
2		Left superior temporal gyrus		-2.1 (1.4)	-1.2 (1.3)
3		Left middle temporal gyrus		-2.8 (1.8)	-1.9 (1.8)
4		Left inferior temporal cortex	Inferior temporal gyrus<br>Fusiform gyrus	-3.5 (2.6)	-2.6 (2.4)
5		Left amygdala		-3.0 (2.3)	-2.9 (2.3)
6		Left parahippocampal gyrus	Parahippocampal gyrus<br>Entorhinal areas	-1.9 (1.7)	-3.0 (2.6)
7		Left hippocampus		-1.9 (1.7)	-1.6 (1.4)
8		Right temporal pole		-1.2 (1.6)	-1.8 (2.0)
9		Right middle temporal gyrus		-1.5 (1.7)	-1.3 (1.7)
10		Right inferior temporal cortex	Inferior temporal gyrus<br>Fusiform gyrus	-1.5 (1.8)	-1.8 (2.4)
11		Right amygdala		-1.6 (1.7)	-1.7 (1.9)
12	Parietal Lobe	Left temporoparietal junction	Supramarginal gyrus<br>Angular gyrus<br>Planum temporale	-1.7 (1.6)	-0.7 (0.8)
13		Left other parietal	Parietal operculum<br>Superior parietal lobule<br>Postcentral gyrus<br>Postcentral gyrus medial segment	-1.2 (1.5)	--0.0 (1.0)
14	Frontal Lobe	Left inferior frontal gyrus		-1.2 (1.5)	-2.5 (2.3)
15		Left other frontal	Superior frontal gyrus<br>Middle frontal gyrus<br>Medial frontal cortex<br>Subcallosal area<br>Superior frontal gyrus medial segment<br>Precentral gyrus medial segment<br>Frontal operculum<br>Central operculum<br>Frontal pole	-1.3 (1.7)	-0.6 (1.6)
16		Left precentral gyrus		-0.9 (1.3)	-0.4 (1.4)

17		Left anterior cingulate		-1.4 (1.7)	-0.8 (1.4)
18	Insula cortex	Left insula	Posterior insula<br>Anterior insula	-2.9 (1.8)	-2.8 (1.7)
19		Right insula	Posterior insula<br>Anterior insula	-1.7 (1.5)	-1.5 (1.8)

Supplementary Table 2: **Regions of interest included in the model.** The 19 regions included in the model, as well as a breakdown of sub regions within them. The w-scores result from standardising the raw volumes (calculated using geodesic information flow (GIF)[1] and the neuromorphometrics brain parcellation) with respect to covariates of: age, sex, total intercranial volume and scanner type. The w-scores for the Queen Square dataset and ALLFTD dataset are calculated with respect to controls from the corresponding dataset.

Model hyperparameters

w-score thresholds

Supplementary Fig. 2 shows the cumulative distribution function of w-scores for each of the 19 regions of interest in the Queen Square dataset. The model used three severity scores (1,2,3) as thresholds of abnormality from the control population.[2] The choice of integer value thresholds aids interpretability. Furthermore, this choice of threshold is in line with previous applications of SuStaIn from the literature.[3] We justified the choice of a maximum w-score threshold of three since the 95th percentile falls beyond a w-score of three in all 19 regions, so at least 5% of the sample have reached the most severe score in every region.[4]

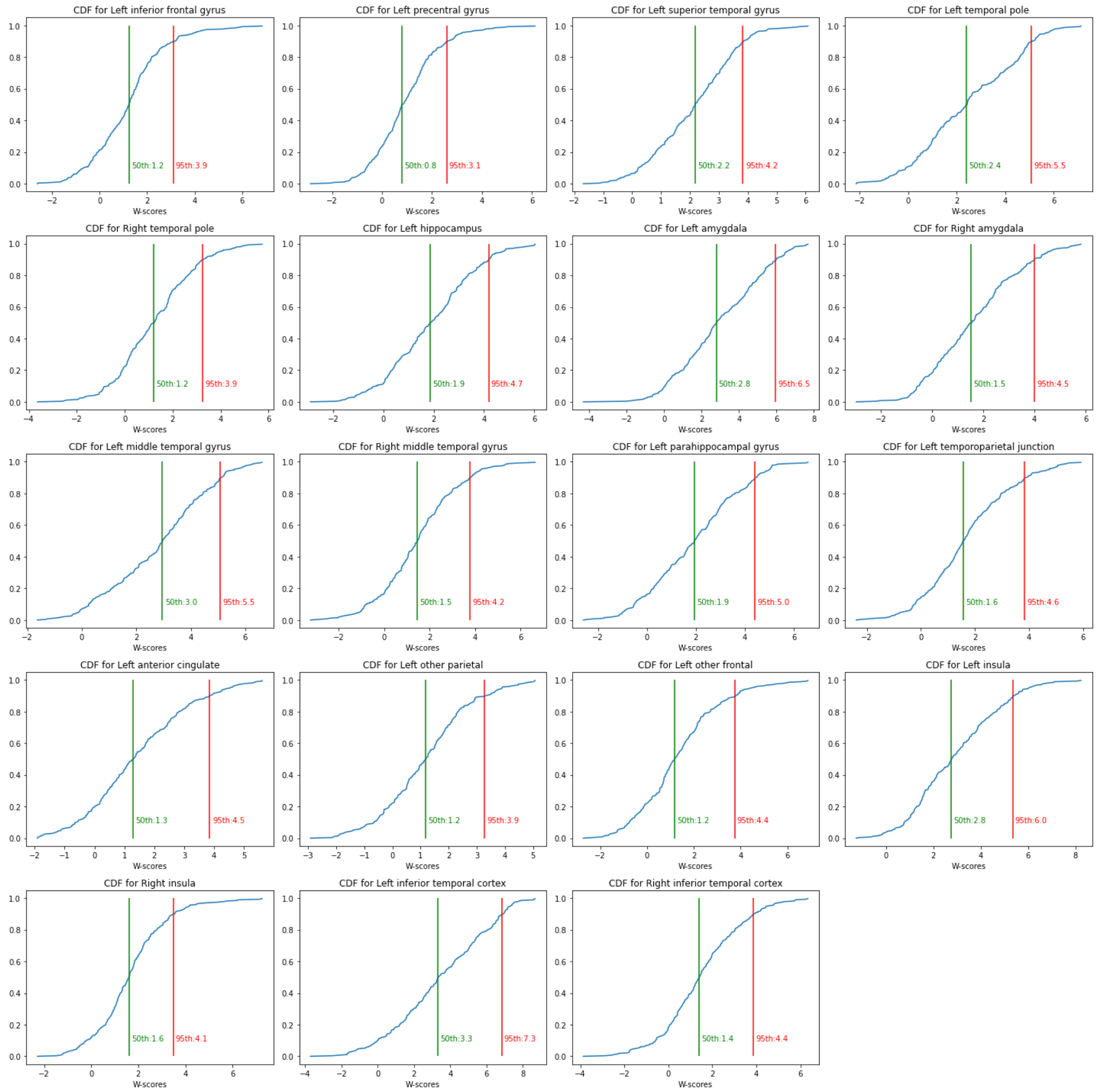

Supplementary Figure 2: **Cumulative distribution function of w-scores across the 19 ROIs** used in the model. Green vertical line represents the 50th percentile of each region (50th: w-score corresponding to 50th percentile), red vertical line represents the 95th percentile of each region (95th: w-score corresponding to 95th percentile).

Model fitting

Supplementary Fig. 3a demonstrates the test set log likelihood across folds, Supplementary Fig. 3b demonstrates the cross-validation information criterion (CVIC) achieved by the model for n subtypes displayed along the x-axis.[2] Higher log-likelihood and lower CVIC represents better model fit.

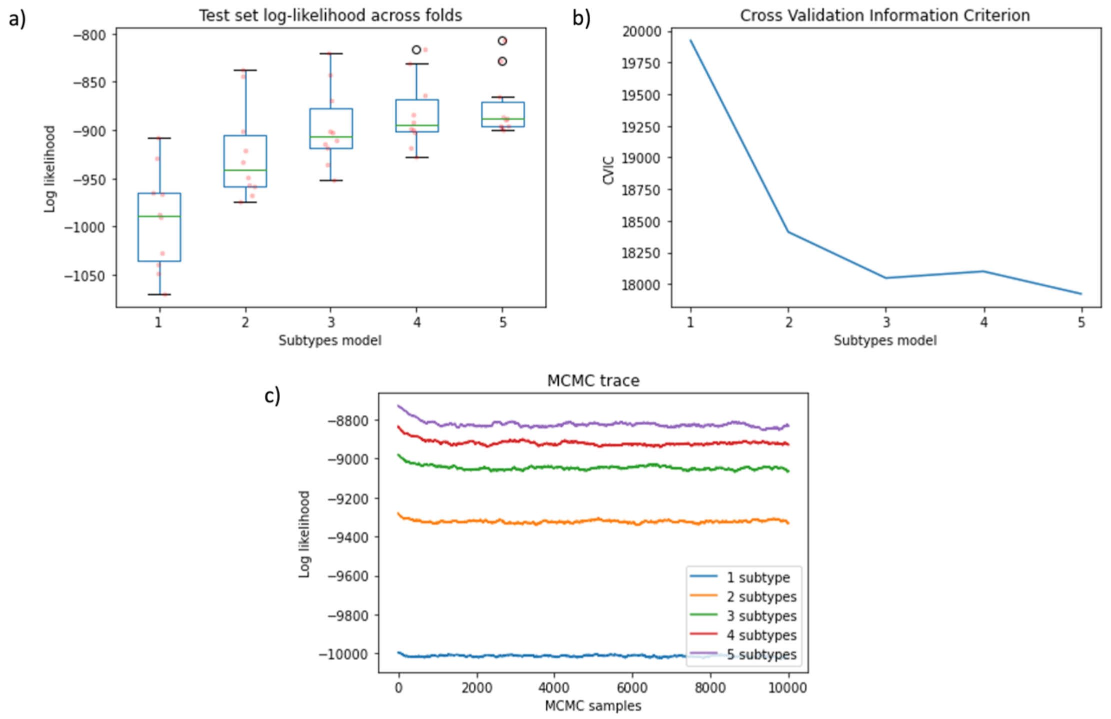

Supplementary Figure 3: **Model fitting and cross-validation.** a) Test set log likelihood across folds b) Cross validation information criterion for n subtype models c) The MCMC trace for the 5-subtype model which was used to select the optimal number of subtypes.

Hellinger distance similarity measure for data-driven subtypes

To have a metric of statistical similarity between positional variance diagrams we calculated a Hellinger distance reference value. Following the framework outlined by Oxtoby *et al*[5] for each of the four model subtypes, we calculated the mean Hellinger distance between a new positional variance diagram (resulting from randomly permuting its rows), and the other three subtypes. For each subtype we repeated this Hellinger distance of permuted positional variance 100 times, the mean across all values was taken to be the reference value $H_0 = 0.89 \pm 0.019$ (95% CI [0.893, 0.895]).

Results: Queen Square discovery set

Stage and subtype assignment

Supplementary Fig. 4 shows the number of individuals assigned each stage by subtype. The model included 57 stages, but no patients were above stage 50, with most patients assigned to early and mid-stages of the disease.

Supplementary Fig. 5 shows the probability with which patients were assigned a subtype according to the choice which maximised their subtype probability.

Supplementary Table 4 shows the paired Hellinger distance between subtypes positional variance diagrams. The low similarity between subtypes supports them representing statistically different sequences of neuroanatomy events.

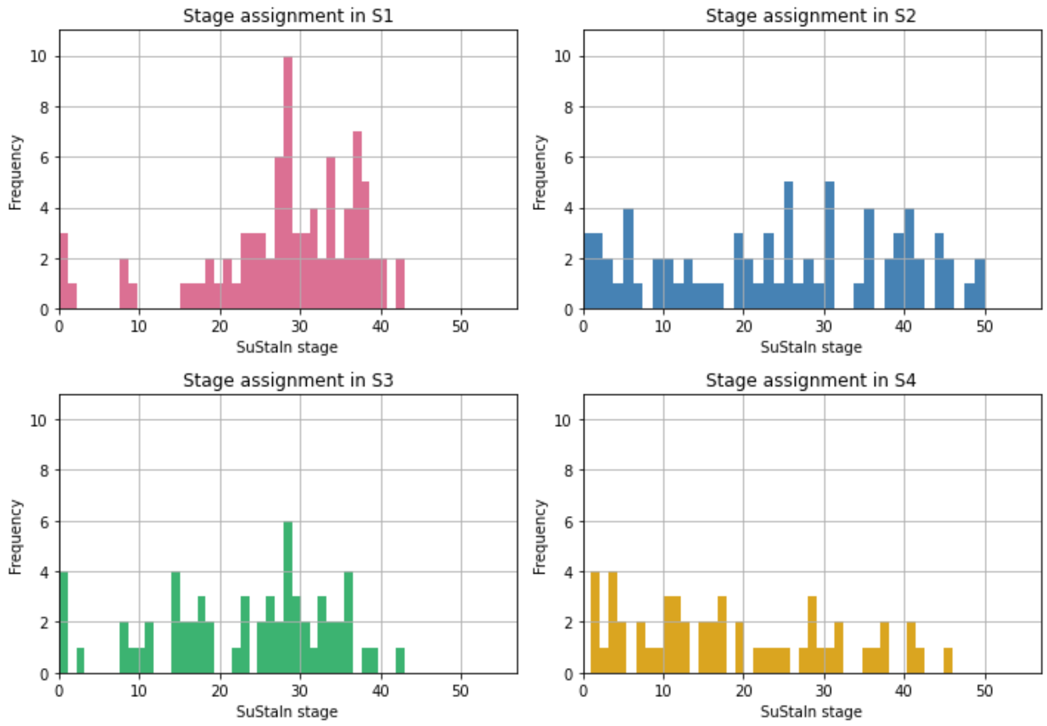

Supplementary Figure 4: **Data-driven stage assignment in the four data-driven subtypes.** Colours are used to distinguish the four subtypes: S1 (pink), S2 (blue), S3 (green), S4 (yellow).

	S1<br>(left temporal)	S2<br>(insula)	S3<br>(temporoparietal)	S4<br>(frontoparietal)
S1<br>(left temporal)	0.0	0.85	0.8	0.78
S2<br>(insula)	0.85	0.0	0.82	0.9
S3<br>(temporoparietal)	0.8	0.82	0.0	0.81
S4<br>(frontoparietal)	0.78	0.9	0.81	0.0

Supplementary Table 4: **Paired Hellinger distances between data-driven subtypes**. A Hellinger distance of 0 indicates perfect agreement between two subtypes posterior event distributions, and 1 indicates complete disagreement between two subtypes posterior event distributions. Colours are used to distinguish the four subtypes: S1 (pink), S2 (blue), S3 (green), S4 (yellow).

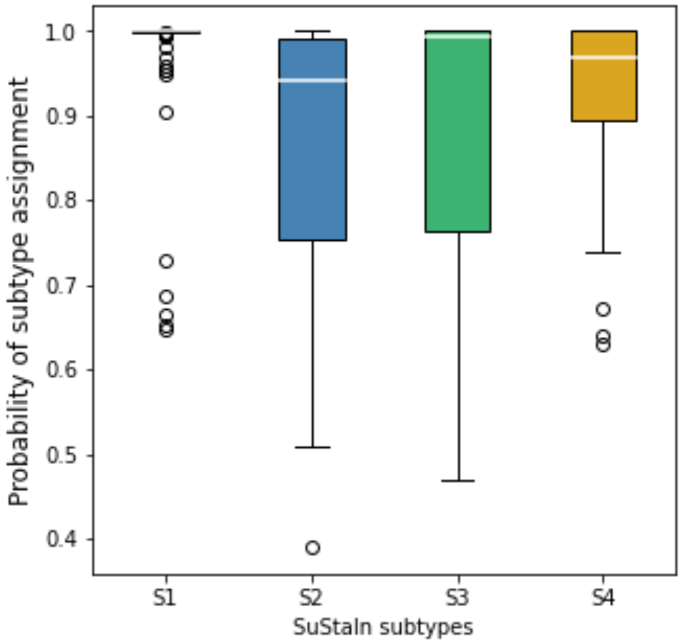

Supplementary Figure 5: **Probability of subtype assignment at baseline across the four subtypes.** The y axis is the baseline probability amongst patients assigned to the respective subtypes at baseline.

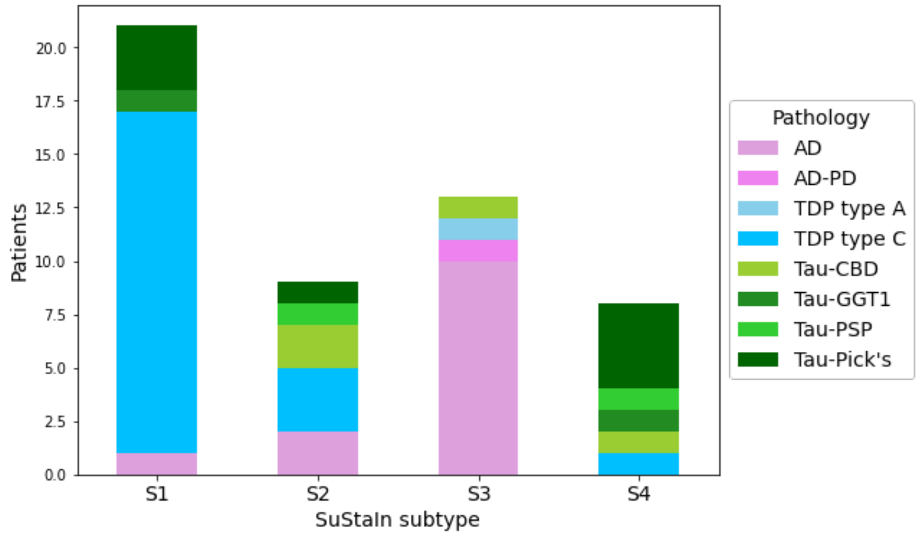

Supplementary Figure 6: **Comparison between data-driven subtype and primary pathology.** Stacked bar chart represents the number of patients with each primary pathology by data-driven subtype.

Abbreviations: AD – Alzheimer's disease, AD-PD – Alzheimer's disease with Parkinsons, TDP type A, TDP – TAR DNA binding protein 43, Tau – FTLD-tau.

Association with clinical phenotypes

Phenotypic disease progression

Supplementary Fig. 7 shows a single disease progression pattern per clinical phenotype. Each disease progression pattern is estimated using SuStaIn (with the subtype parameter set to one – i.e. a fixed single 'subtype') with data from a single phenotype.[2,6]

Supplementary Fig. 8 shows the single disease progression pattern per clinical phenotype, where the model complexity has been reduced, down to a single normal/abnormal w-score event. Each disease progression pattern is estimated using SuStaIn (with a single 'subtype') with data from a single phenotype, but this time using just one w-score of 1. The sequence of ROIs becoming abnormal is very similar to the three w-score model (Supplementary Fig. 7). The one-w-score model offers a clearer visualisation of disease progression, which we subsequently utilise for analysis in Supplementary Fig. 9.

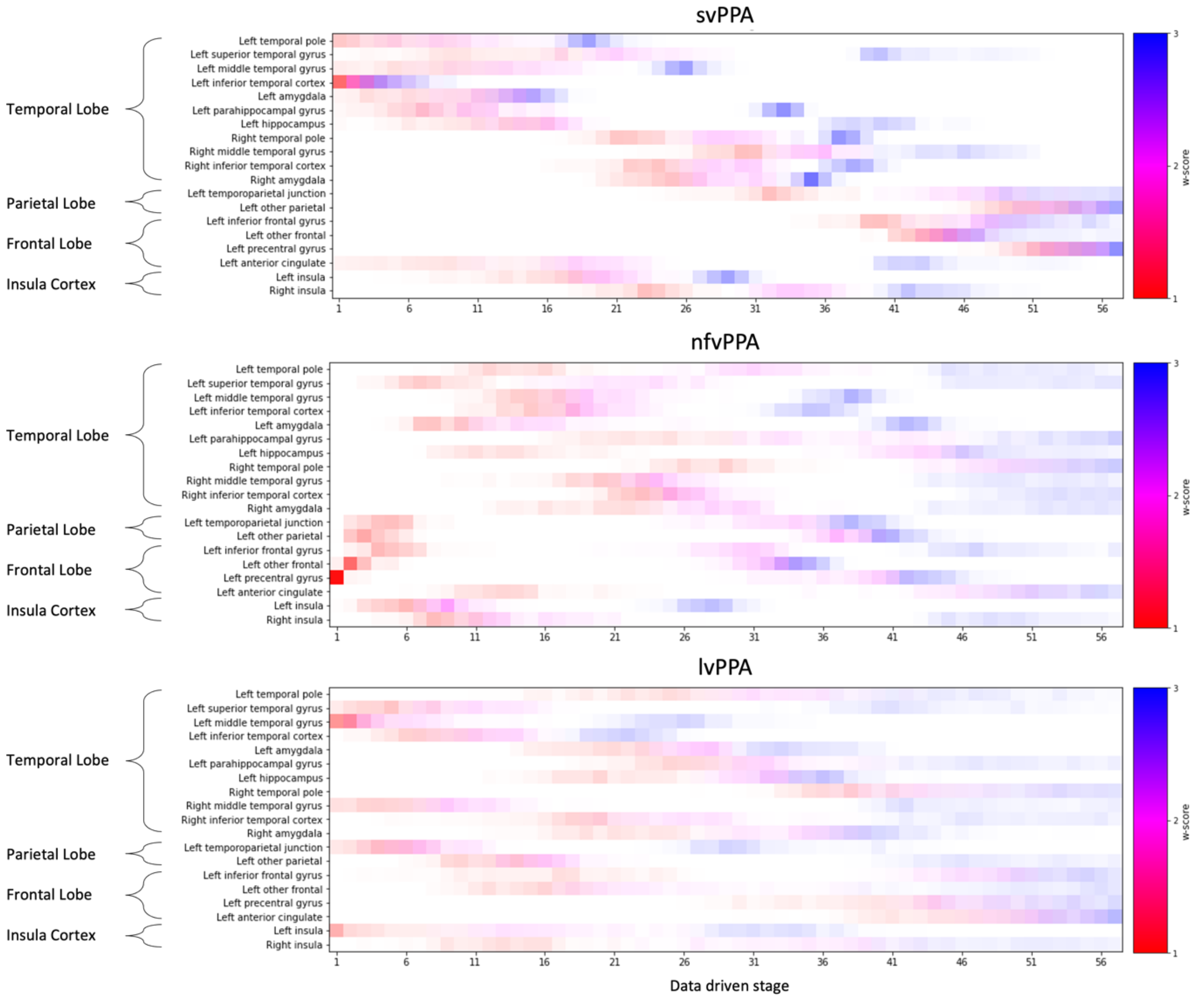

Supplementary Figure 7: **Positional variance diagrams for phenotypic data.** Along the y-axis are the regions of interest used in the model, grouped by location in the brain. The data-driven stages correspond to the sequence that brain regions become abnormal, with colour representing degree of abnormality (w-score 1: red, w-score 2: pink, w-score 3: blue), and colour density representing model certainty.

Abbreviations: svPPA – semantic variant PPA, nfvPPA – non-fluent/agrammatic variant PPA, lvPPA – logopenic variant PPA.

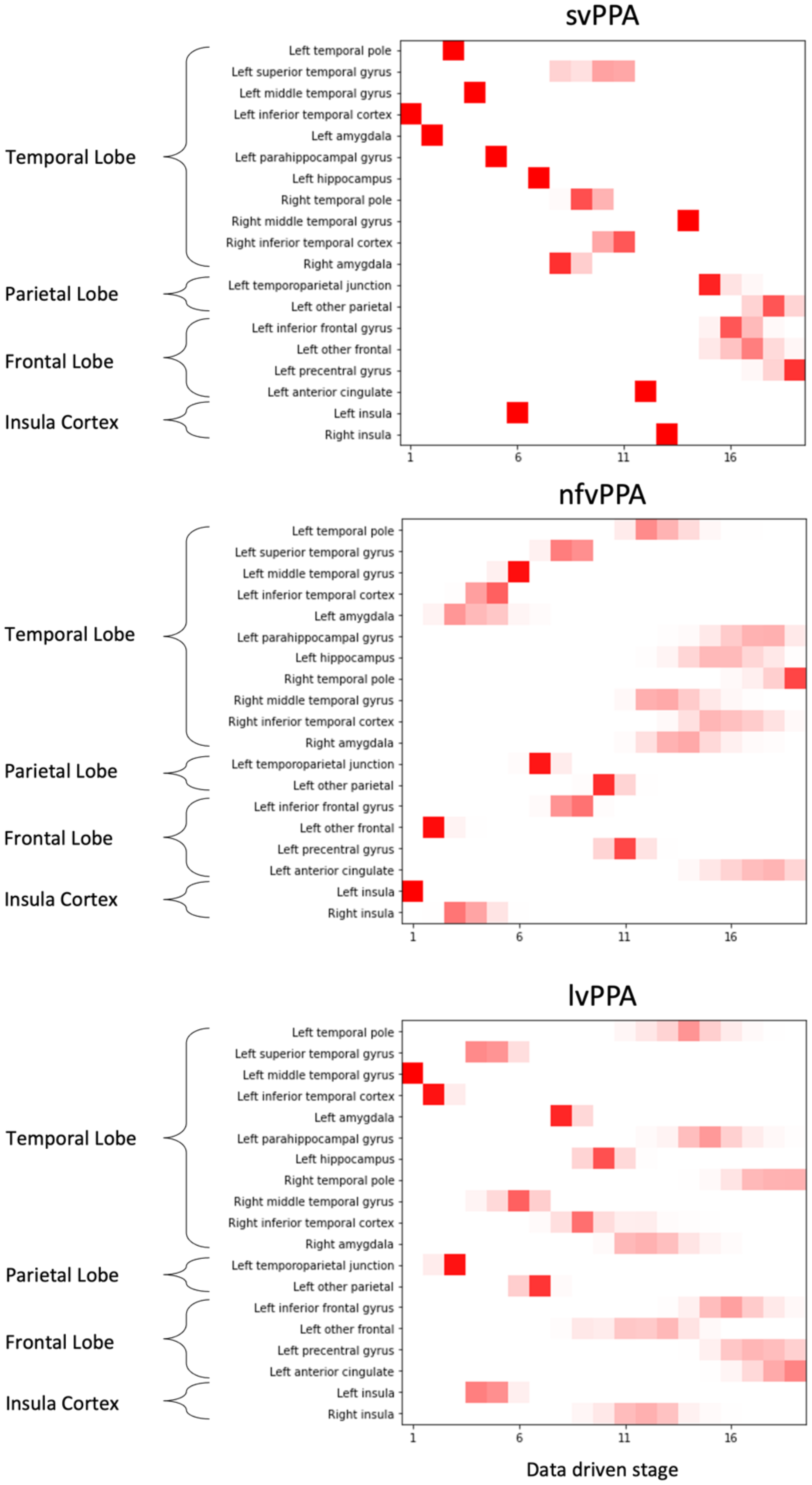

Supplementary Figure 8: **Positional variance diagrams for phenotypic data with one w-score.** Along the y-axis are the regions of interest used in the model, grouped by location in the brain. The data-driven stages correspond to the sequence that brain regions become abnormal, with colour density representing model certainty.

Abbreviations: SuStaIn – Subtype and Stage Inference algorithm, svPPA – semantic variant PPA, nfvPPA – non-fluent/agrammatic variant PPA, lvPPA – logopenic variant PPA.

Phenotypic disease progression vs. Subtype disease progression

Supplementary Table 6 records the paired Hellinger distances between the four data-driven subtypes and the three clinical phenotypes.

Supplementary Fig. 9 shows the sequence that ROIs become abnormal in each subtype compared to the clinical phenotypes. The sequences of ROI progression in the data-driven subtypes were extracted from the three w-score positional variance diagrams by calculating at which stage each event reached w-score=1 with a probability of 0.75. These stages were then used to order the events sequentially. The sequences were calculated similarly in the clinical phenotypes, but directly from the one w-score model. In the subplots a/b/c/d the x-axis is ordered according to the sequence of disease progression in S1/S2/S3/S4 respectively. Each clinical phenotype is then plotted as a connected line denoting when each event occurred. Complete agreement between sequence of ROIs in the subtype and clinical phenotype would follow the identity line. In the figure we highlight the sequences which were statistically correlated according to the Hellinger distance analysis (Supplementary Table 6): S1 and svPPA (shown in bold pale pink in Supplementary Fig. 9a); S3 and lvPPA (shown in bold dark green in Supplementary Fig. 9c).

		**Data-driven subtype**			
		S1 (left temporal)	S2 (insula)	S3 (temporoparietal)	S4 (frontoparietal)
**Clinical phenotype**	svPPA	0.44	0.77	0.85	0.90
	nfvPPA	0.86	0.75	0.74	0.60
	lvPPA	0.82	0.73	0.46	0.75

Supplementary Table 6: **Hellinger distances between phenotype models and data-driven subtypes.** The Hellinger distance between the posterior identified for each clinical diagnosis compared to the posterior identified for the four data-driven subtypes. Colours are used to distinguish the four subtypes: S1 (pink), S2 (blue), S3 (green), S4 (yellow).

Abbreviations: svPPA – semantic variant PPA, nfvPPA – nonfluent/agrammatic variant PPA, lvPPA – logopenic variant PPA.

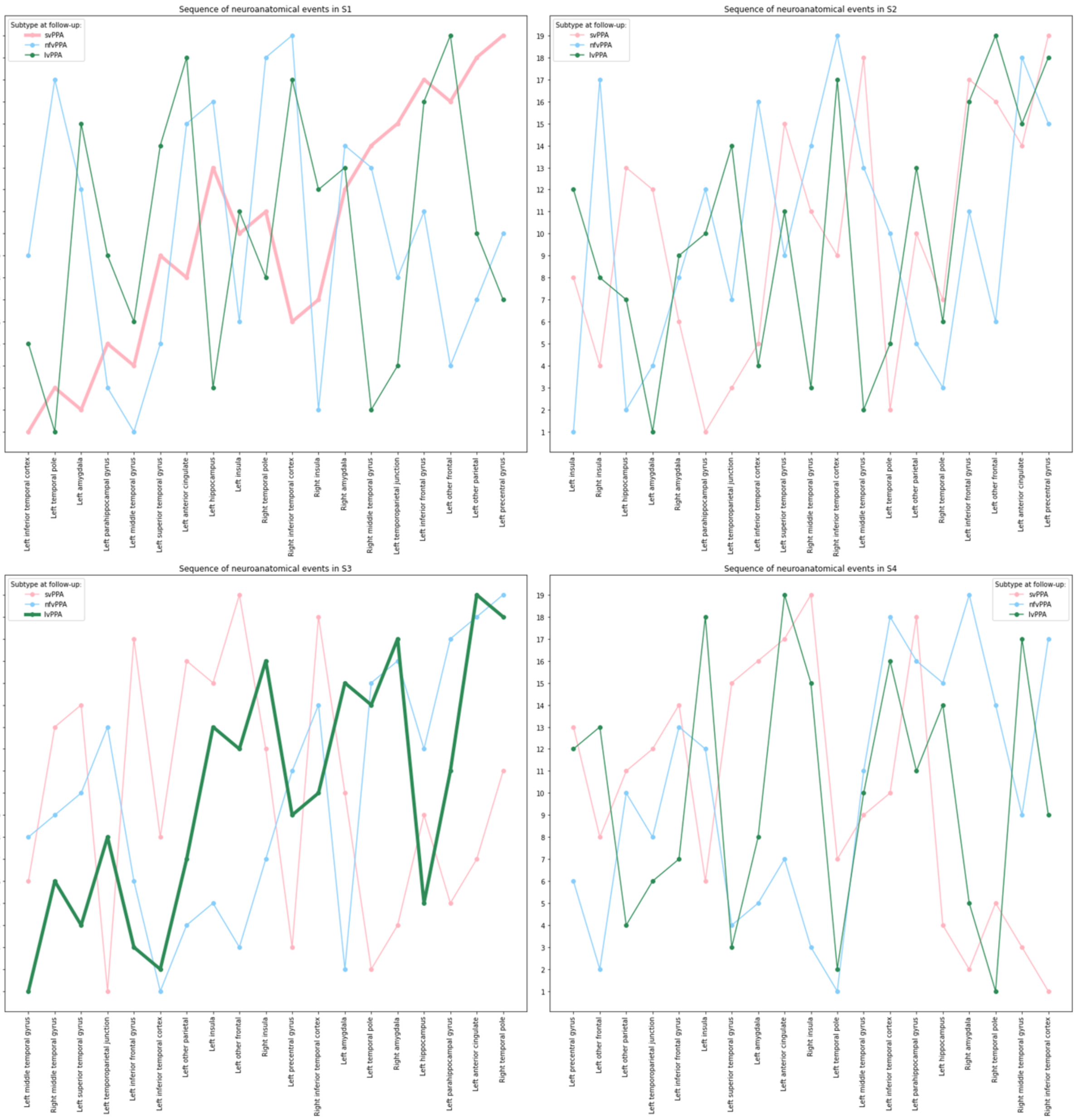

Supplementary Figure 9: **Sequence of neuroanatomical pathology: data-driven subtypes vs. phenotypes.** Along the x-axis are the regions of interest used in the model, ordered according to the sequence of neuroanatomical pathology per subtype, a) S1 left temporal, b) S2 right, c) S3 temporoparietal, d) S4 frontoparietal. The y-axis is the sequence of neuroanatomical pathology in the clinical phenotypes. Colours are used to distinguish the three phenotypes: svPPA (pale pink), nfvPPA (pale blue), lvPPA (dark green).

Abbreviations: svPPA – semantic variant PPA, nfvPPA – non-fluent/agrammatic variant PPA, lvPPA – logopenic variant PPA.

Longitudinal analysis

Supplementary Fig. 10a shows the distribution of subtype assignment probabilities at baseline stratified by whether patients changed subtype assignment at first follow up. Supplementary Fig. 10b shows the distribution of subtype assignment probabilities at baseline stratified by whether patients regressed or progressed/were stable at first follow up.

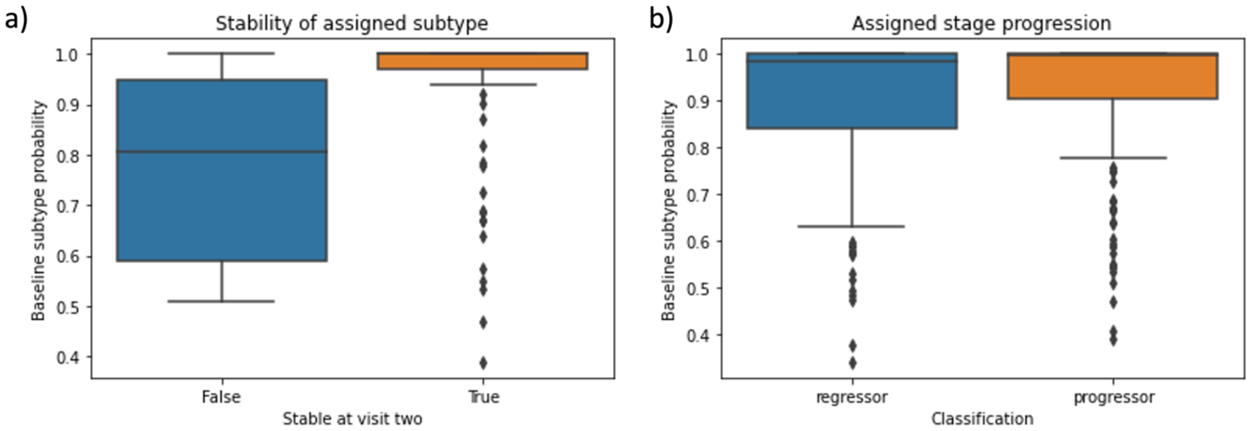

Supplementary Figure 10: a) **Subtype consistency at first follow up visit**. The y-axis shows the baseline subtype probability for the subtype individuals were assigned to, so a value of one suggests high confidence in the subtype assignment, whilst a value nearer to 0 suggests lower confidence. The x-axis labels correspond to whether patients assigned subtype changed at first follow up (False), or stayed the same (True). b) **Staging consistency at first follow up**. The y-axis shows the baseline subtype probability for the subtype individuals were assigned to, so a value of one suggests high confidence in the subtype assignment, whilst a value nearer to 0 suggests lower confidence. The x-axis labels correspond to whether patients assigned stage was consistent at first follow up (progressor – they progressed or remained stable) or inconsistent (they regressed).

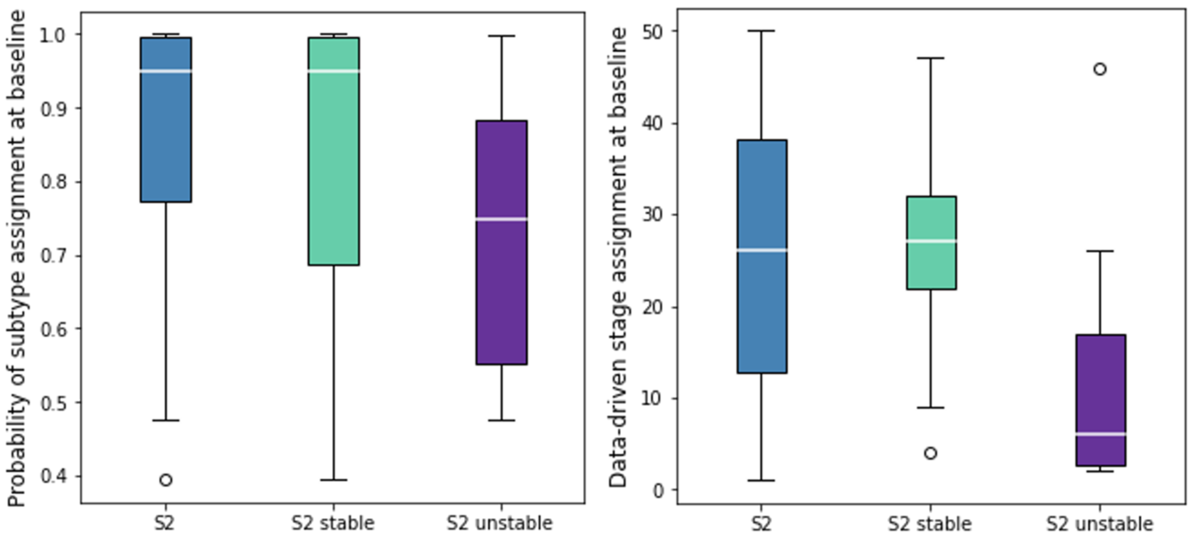

Supplementary Figure 11: **Stability of S2 (insula).** A breakdown of the baseline probability of subtype assignment (left), and baseline stage assignment (right) in S2.

W-score sensitivity analysis

To check the sensitivity of the model to the choice of w-scores, w=(1,2,3), we re-ran the SuStaIn algorithm using alternative w-score thresholds.

Supplementary Fig. 12 and Supplementary Fig. 13 shows the results of the model using two w-score thresholds of w=(1,3). The cross-validation information criterion (Supplementary Fig. 12) supports the identification of four subtypes. From the positional variance diagrams in Supplementary Fig. 13 these four subtypes correspond to those identified in the original analysis, with the same left temporal, insula, temporoparietal and frontoparietal patterns present.

Supplementary Fig. 14 and Supplementary Fig. 15 shows the results of the model using three alternative w-score thresholds of w=(2,4,5). Again, the cross-validation information criterion (Supplementary Fig. 14) supports the identification of four subtypes. Similarly to the two threshold model, the four subtypes correspond to those identified in the original analysis, with the same left temporal, insula, temporoparietal and frontoparietal patterns present (Supplementary Fig. 15).

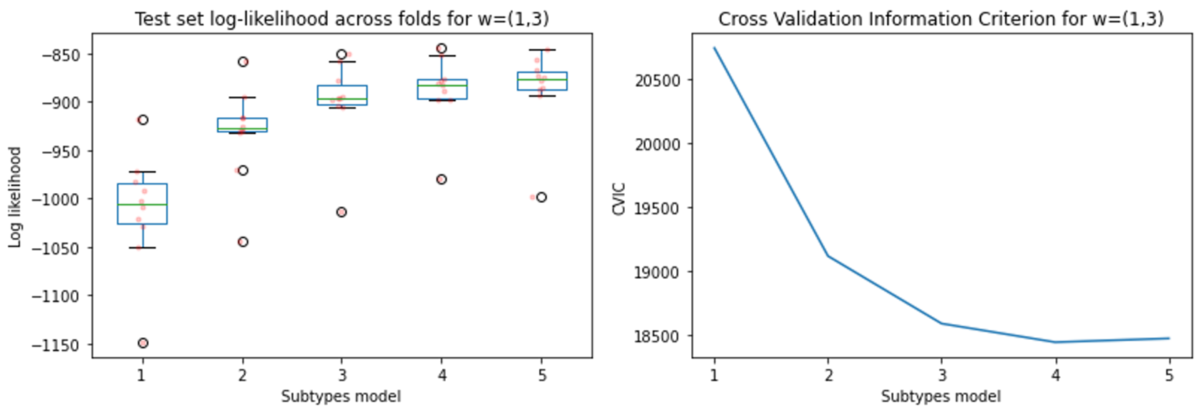

Supplementary Figure 12: **(left) Test set log likelihood across folds (right) Cross validation information criterion for n subtype models.**

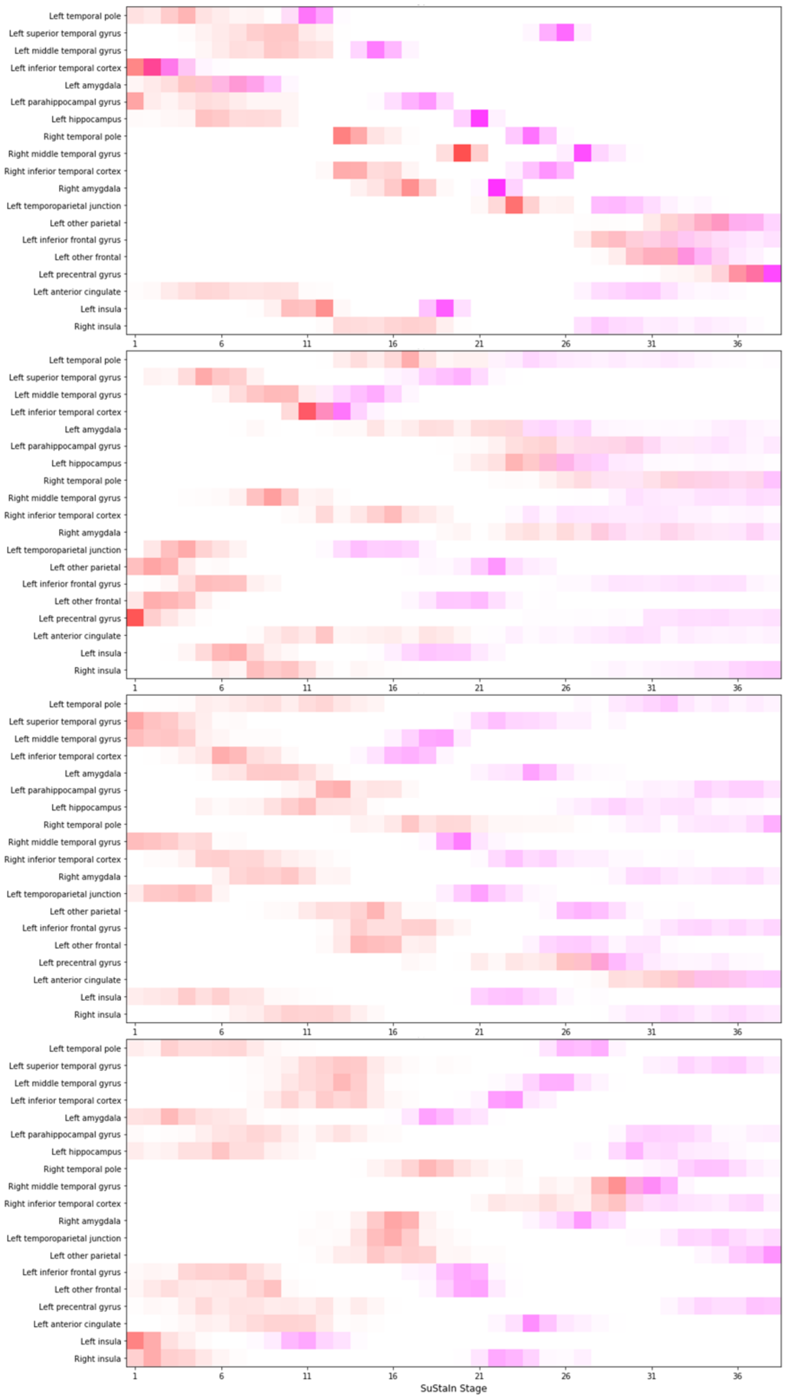

Supplementary Figure 13: **Positional Variance Diagrams for the four subtypes identified by the model with w-score thresholds = (1,3).**

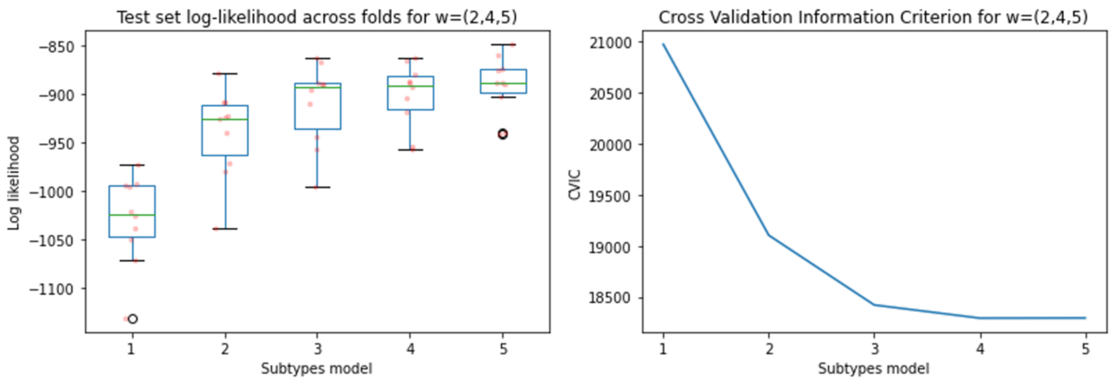

Supplementary Figure 14: **(left) Test set log likelihood across folds (right) Cross validation information criterion for n subtype models.**

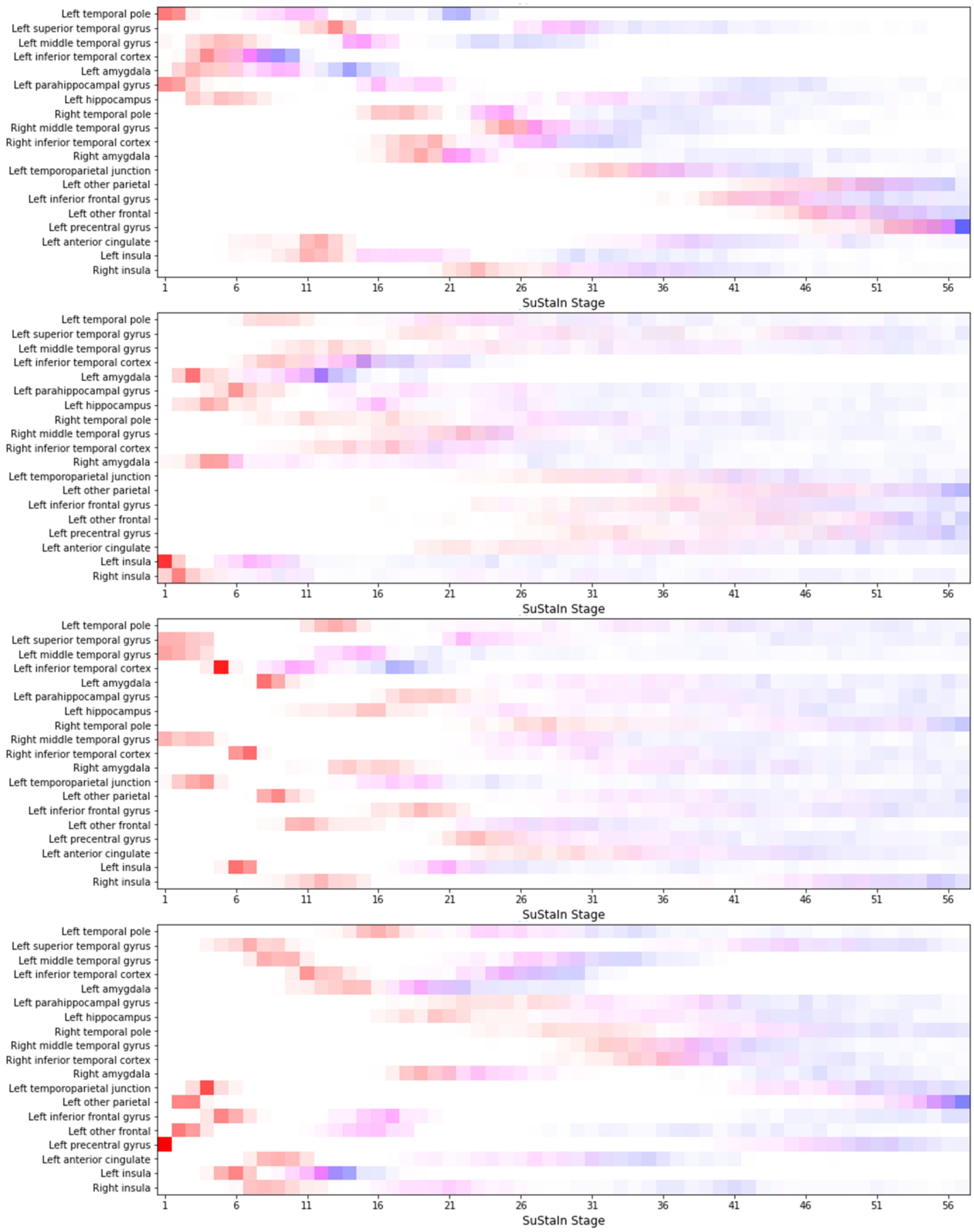

Supplementary Figure 15: **Positional Variance Diagrams for the four subtypes identified by the model with w-score thresholds = (2,4,5).**

Longitudinal analysis of stable scanner type

Since 22% (30/137) of individuals changed scanner manufacture or scanner strength longitudinally we repeated the longitudinal analysis restricted to those who were scanned on the same scanner at all time points.

Of the 107 individuals with longitudinal data collected on the same scanner, 105 were deemed subtypable as they were assigned above stage 0 at baseline. Supplementary Fig. 16a shows longitudinal consistency of model subtype assignments for these 105 patients. Subtype consistency between first and second MRI scan was: 89.5/60.7/95.2/94.4% for S1/S2/S3/S4 respectively. Of the 88/105 (83.8%) of patients whose subtype assignment was stable at the first two timepoints, the mean probability of baseline subtype assignment was 0.93.

Supplementary Fig. 16b and 16c show longitudinal consistency of model staging, stratified by model subtype (b) and clinical diagnosis (c). At first follow up, 87/105 (82.9%) patients advanced (upward) to a later stage, 12/105 (11.4%) patients remained at the same stage (on the diagonal), while a further 6/105 (5.7%) patients regressed (downward) to an earlier stage, giving a longitudinal staging consistency exceeding 94.3% (99/105).

Of the 42 patients having three or more MRI scans and subtypable at baseline, 28 were assigned the same subtype at every timepoint, and 33 were assigned monotonically increasing stages across the timepoints.

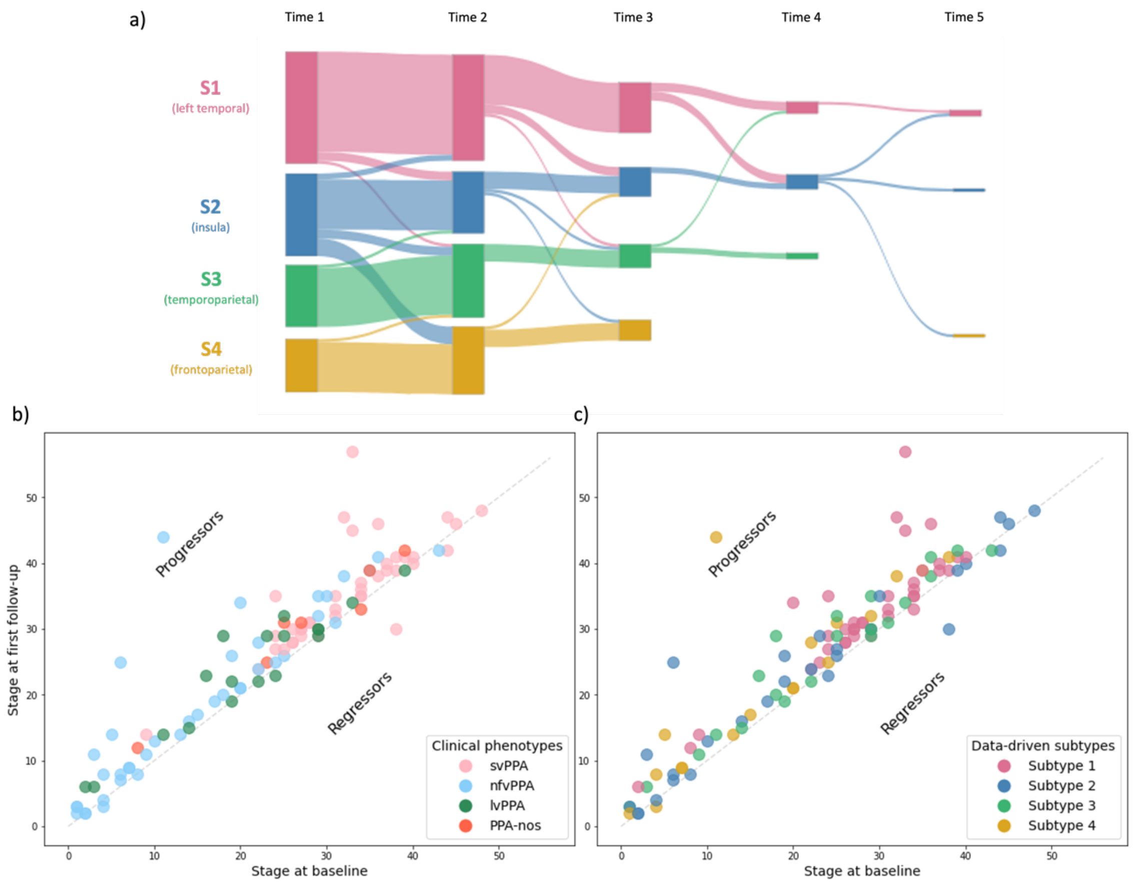

Supplementary Figure 16: **a) Sankey diagram of subtype assignment between baseline and first follow-up clinic visit (stable scanner).** The bars are colour coded to represent the percentage of patients in each subtype at first follow up, stratified by their subtype assignment at baseline S1 anterior temporal (pink), S2 insula (blue), S3 TPJ (green), S4 precentral gyrus (yellow). The data was restricted to just those individuals at each time who had been scanned on the same type of scanner up to and including that time. **Data-driven stage assignment at baseline and first follow up.** Patients are colour coded according to clinical diagnosis (b) and according to subtype assignment (c). Those above the diagonal progressed at follow up, whilst those below regressed.

Abbreviations: svPPA – semantic variant PPA, nfvPPA – nonfluent/agrammatic variant PPA, lvPPA – logopenic variant PPA, PPA-nos – PPA not otherwise specified.

Post 2010 Dataset

The diagnostic term of lvPPA was enshrined in consensus criteria in 2011.[7,8] Despite the re-diagnosis of pre-2010 cases by a senior neurologist we were cautious that they were more susceptible to misdiagnosis. To investigate this we re-ran the analysis on a subset of the data,

restricted to those individuals diagnosed at Queen Square in or after 2011. The demographics of the post 2010 subset is given in Supplementary Table 7.

Post 2010 demographics

		PPA	svPPA	nfvPPA	lvPPA	PPA-nos	Controls
		Queen Square discovery dataset (post 2010)					
n		129	37	52	28	12	121
Sex		60F:69M	16F:21M	29F:23M	10F:18M	5F:7M	65F:56M
Age at onset, years, mean ± SD		62.9±7.9	55.3±6.7	65.6±8.3	63.5±7.6	59.9±5.9	-
Age at baseline scan, years, mean ± SD		67.2±7.8	64.3±6.5	69.6± 8.3	68.2±7.2	63.1±5.8	61.7± 11.1
Primary pathology, n							
	Alzheimer's disease	2	0	0	2	0	-
	FTLD-tau	6	0	4	0	2	-
	FTLD-TDP43	3	1	2	0	0	-
Secondary clinical diagnosis, n							
	PD	0	0	0	0	0	-
	PSP	3	0	3	0	0	-
	CBS	5	0	4	0	1	-
	PSP/CBS	1	0	1	0	0	-
	MND	0	0	0	0	0	-

Supplementary Table 7: **Post 2010 cohort demographics.** Colours are used to distinguish the three phenotypes: svPPA (pale pink), nfvPPA (pale blue), lvPPA (dark green). Abbreviations: PPA – Primary Progressive Aphasia, svPPA – semantic variant PPA, nfvPPA – nonfluent/agrammatic variant PPA, lvPPA – logopenic variant PPA, PPA-nos – PPA not otherwise specified, FTLD-tau – Frontotemporal lobal degeneration tau, FTLD-TDP43 – frontotemporal lobar degeneration TAR DNA-binding protein 43, PD – Parkinson's disease, PSP – progressive supranuclear palsy, CBS – corticobasal syndrome, MND – motor neurone disease.

Post 2010 results

We used the same ROIs, and same parameters as for the discovery dataset model. Model cross-validation found 2 subtypes to be optimal in this subset. When we forced the model subtype hyperparameter to be four, the four subtypes were similar to those in the discovery cohort.

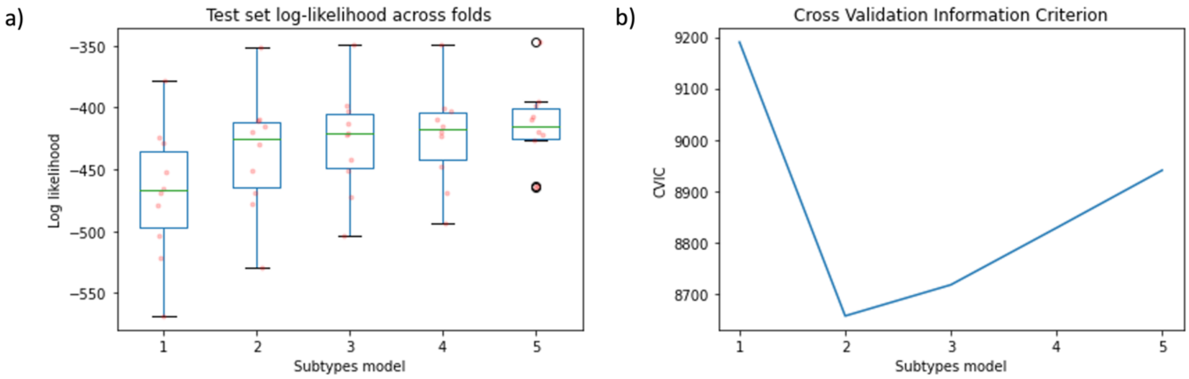

Supplementary Figure 17: a) Test set log likelihood across folds (post 2010 subset) b) Cross validation information criterion for n subtype models (post 2010 subset).

Two subtype model

In the two subtype model the first subtype (S1 - left precentral gyrus) there was initial marked atrophy in the left precentral gyrus, followed by parietal lobe and frontal lobe regions. This was followed by diffuse atrophy of the temporal lobe and insula cortex.

In the second subtype (S2 – left insula) there was initially rapid progression through the first and second w-scores in the left insula. This was followed by rapid brain volume change in the left amygdala and the left inferior temporal cortex. There was relative sparing of the frontal lobe and parietal lobe.

In the two subtype model 127/129 patients were assigned above stage 0, and hence deemed to be subtypable, the demographics by subtype are given in Supplementary Table 8. Supplementary Fig. 19 compared clinical phenotypes of those assigned to the two subtypes; S2 (left insula) was largely associated with svPPA, whilst S1 (left precentral gyrus) was a mix of nfvPPA and lvPPA.

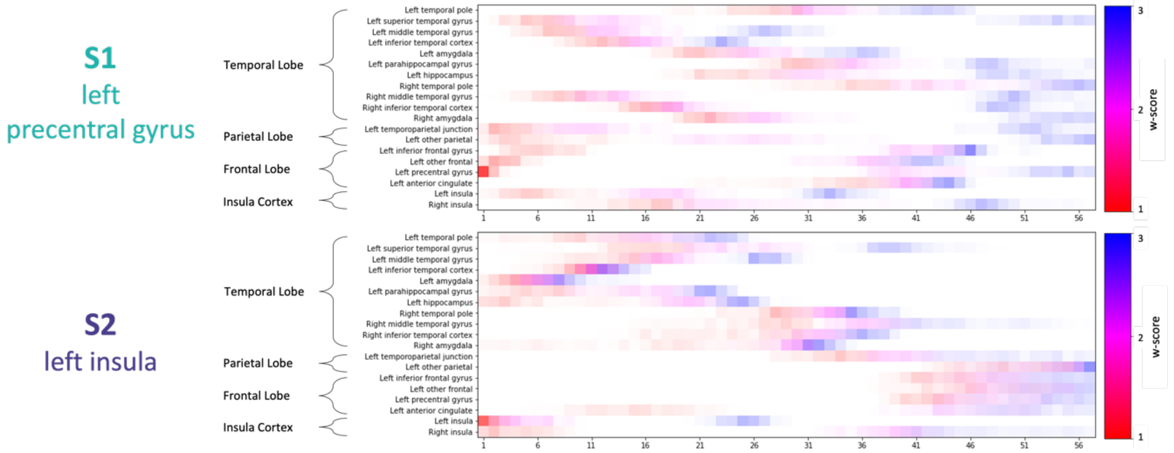

Supplementary Figure 18: **Positional variance diagrams for the two data-driven subtypes in the post 2010 subset.** Along the y-axis are the regions of interest used in the model, grouped by location in the brain. The data-driven stages correspond to the sequence that brain regions become abnormal, with colour representing degree of abnormality (w-score 1: red, w-score 2: pink, w-score 3: blue), and colour density representing model certainty.

		S1 (left precentral gyrus)	S2 (left insula)
		Queen Square discovery dataset (post 2010)	
n		77	50
Sex		35F:42M	23F:27M
Age at onset, years, mean ± SD		63.5±8.0	61.5±7.3
Age at baseline scan, years, mean ± SD		68.1±8.0	65.6±6.9
Diagnosis, n			
	svPPA	6	31
	nfvPPA	42	8
	lvPPA	24	4
	PPA-nos	5	7
Primary pathology, n			
	Alzheimer's disease	2	0
	FTLD-tau	3	3
	FTLD-TDP43 type C	1	2
Secondary diagnosis, n			
	PD	0	0
	PSP	3	0
	CBS	3	2
	PSP/CBS	1	0
	MND	0	0

Supplementary Table 8: **Post 2010 subtype demographics in two subtype model.** Colours are used to distinguish the four data driven subtypes: S1 (pink), S2 (blue), S3 (green), S4 (yellow). Abbreviations: PPA – Primary Progressive Aphasia, svPPA – semantic variant PPA, nfvPPA – nonfluent/agrammatic variant PPA, lvPPA – logopenic variant PPA, PPA-

nos – PPA not otherwise specified, FTLD-tau – Frontotemporal lobal degeneration tau, FTLD-TDP43 – frontotemporal lobar degeneration TAR DNA-binding protein 43, PD – Parkinson's disease, PSP – progressive supranuclear palsy, CBS – corticobasal syndrome, MND – motor neurone disease.

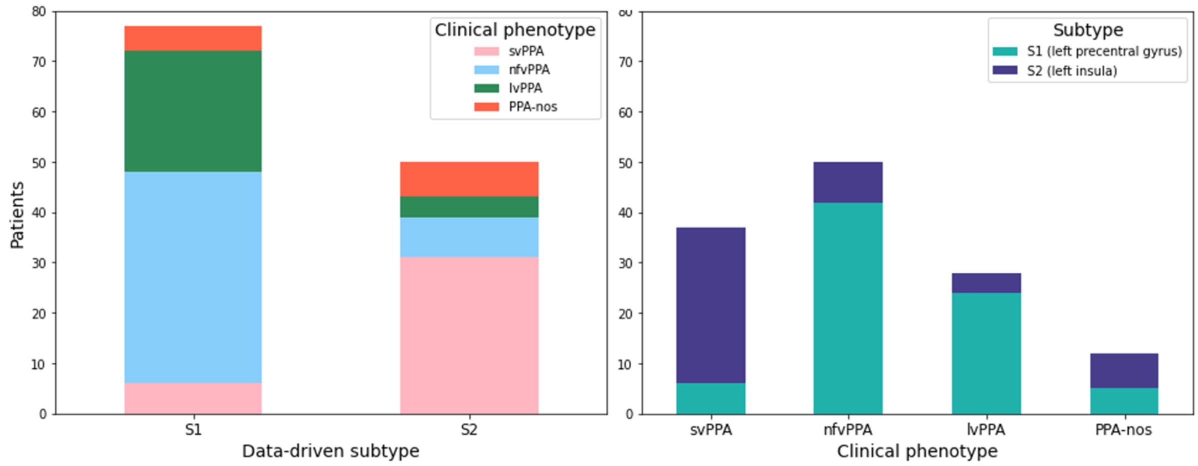

Supplementary Figure 19: **Post 2010 2 subtype model - comparison between data-driven subtype assignment and clinical diagnosis.** The stacked bar chart on the left shows the number of patients with each clinical diagnosis by data-driven subtype assignment. The figure on the right shows the number of patients who were assigned each data-driven subtype by clinical diagnosis.

Abbreviations: svPPA – semantic variant PPA, nfvPPA – nonfluent/agrammatic variant PPA, lvPPA – logopenic variant PPA, PPA-nos – PPA not otherwise specified.

Four subtype model

When constrained to four subtypes, the subtypes resembled those found in the Queen Square discovery dataset. In the four subtype model the positional variance diagrams were largely similar to those seen in the full Queen Square discovery dataset. Visually compared to the positional variance diagrams for the full Queen Square dataset, the four subtypes had higher positional variance, likely due to the lower sample size.

In the four subtype model 127/129 patients were assigned above stage 0, and hence deemed to be subtypable, their demographics are given in Supplementary Table 9. Supplementary Fig. 21 compared clinical phenotypes of those assigned to the four subtypes; similarly to the

full dataset analysis there was a clear correspondence between S1 and svPPA, with more mixed association between S2/S3/S4 and lvPPA and nfvPPA.

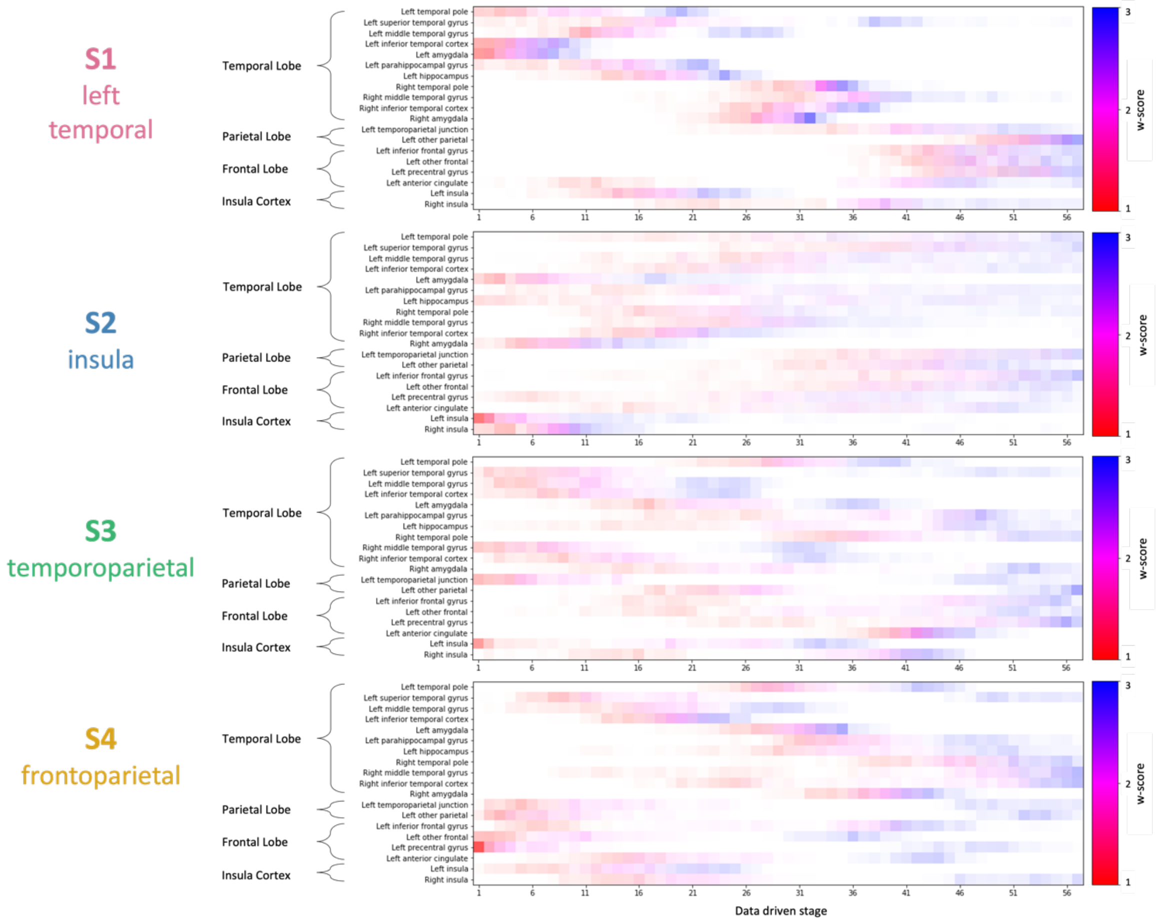

Supplementary Figure 20: **Positional variance diagrams for the four data-driven subtypes in the post 2010 subset.** Along the y-axis are the regions of interest used in the model, grouped by location in the brain. The data-driven stages correspond to the sequence that brain regions become abnormal, with colour representing degree of abnormality (w-score 1: red, w-score 2: pink, w-score 3: blue), and colour density representing model certainty.

	**S1 (left temporal)**	**S2 (insula)**	**S3 (temporoparietal)**	**S4 (frontoparietal)**
	**Queen Square discovery dataset (post 2010)**			
n	42	9	42	34
Sex	19F:23M	4F:5M	20F:22M	15F:19M

Age at onset, years, mean ± SD	60.8±7.0	64.8±7.0	60.8±7.2	67.1±8.0
Age at baseline scan, years, mean ± SD	64.9±6.5	68.3±7.3	65.1±6.8	71.9±8.2
Diagnosis, n				
svPPA	30	1	3	3
nfvPPA	5	6	15	24
lvPPA	1	1	22	4
PPA-nos	6	1	2	3
Primary pathology, n				
Alzheimer's disease	0	0	2	0
FTLD-tau	2	1	0	3
FTLD-TDP43 type C	2	0	0	1
Secondary diagnosis, n				
PD	0	0	0	0
PSP	0	1	1	1
CBS	1	1	0	2
PSP/CBS	0	0	0	1
MND	0	0	0	0

Supplementary Table 9: **Post 2010 subtype demographics in four subtype model.** Colours are used to distinguish the four data driven subtypes: S1 (pink), S2 (blue), S3 (green), S4 (yellow). Abbreviations: PPA – Primary Progressive Aphasia, svPPA – semantic variant PPA, nfvPPA – nonfluent/agrammatic variant PPA, lvPPA – logopenic variant PPA, PPA-nos – PPA not otherwise specified, FTLD-tau – Frontotemporal lobal degeneration tau, FTLD-TDP43 – frontotemporal lobar degeneration TAR DNA-binding protein 43, PD – Parkinson's disease, PSP – progressive supranuclear palsy, CBS – corticobasal syndrome, MND – motor neurone disease.

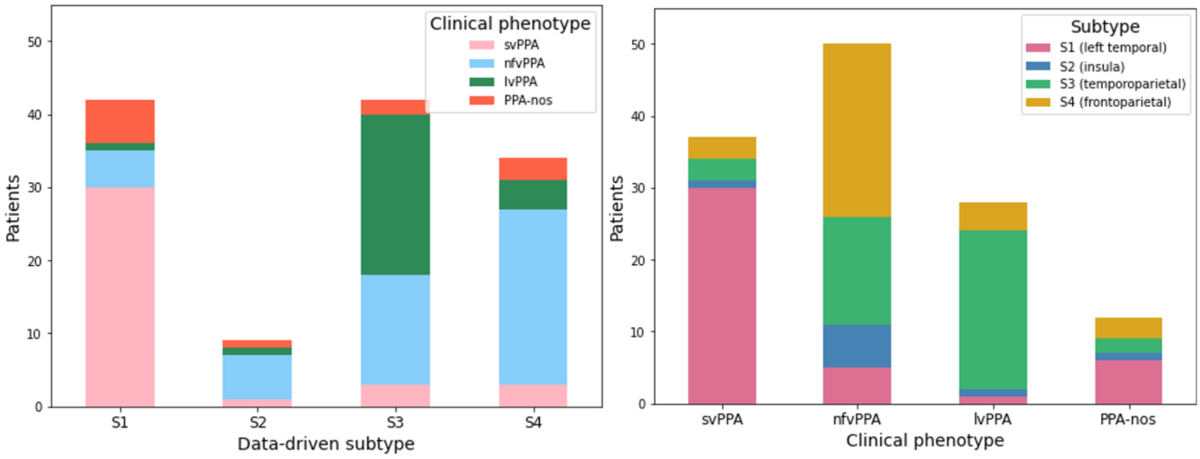

Supplementary Figure 21: **Post 2010 four subtype model - comparison between data-driven subtype assignment and clinical diagnosis.** The stacked bar chart on the left shows the number of patients with each clinical diagnosis by data-driven subtype assignment. The figure on the right shows the number of patients who were assigned each data-driven subtype by clinical diagnosis.

Abbreviations: svPPA – semantic variant PPA, nfvPPA – nonfluent/agrammatic variant PPA, lvPPA – logopenic variant PPA, PPA-nos – PPA not otherwise specified.

Post-2010 summary

This reanalysis aimed to investigate how the re-diagnosis of earlier cases might have influenced the outcomes. Cross-validation of the model indicated that two subtypes were optimal in the post-2010 dataset — likely due to the considerably reduced sample size of the post-2010 dataset (n=129 of 270), and subset class imbalance across diagnoses (40.3% of the subset were diagnosed with nfvPPA), compared to the full dataset. However, the four-subtype model in the post-2010 dataset closely resembled that of the entire discovery dataset, supporting the stability of the four subtypes in the Queen Square discovery dataset.

Results: ALLFTD test set

ALLFTD association with clinical phenotypes

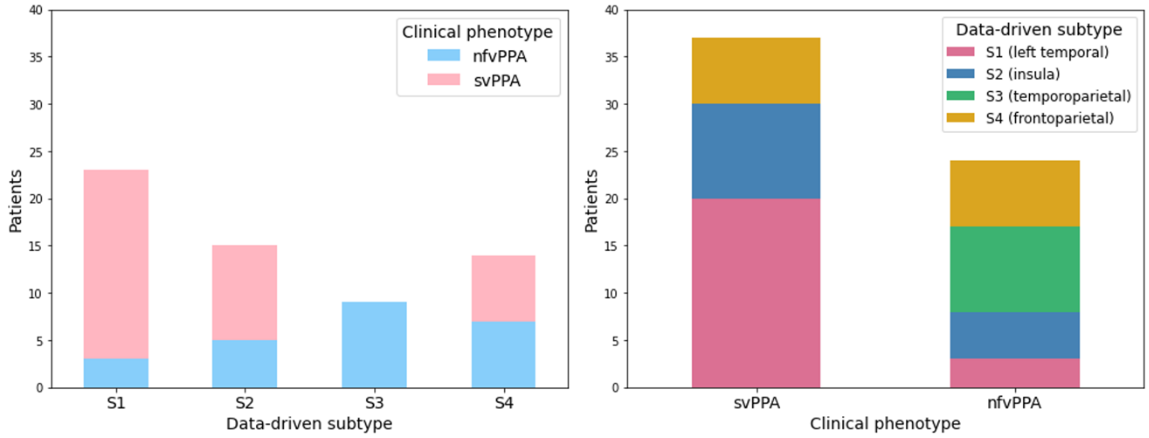

Supplementary Figure 22: **Comparison between data-driven subtype assignment and clinical diagnosis in ALLFTD dataset.** The stacked bar chart on the left shows the number

of patients with each clinical diagnosis by data-driven subtype assignment. The figure on the right shows the number of patients who were assigned each data-driven subtype by clinical diagnosis.
Abbreviations: svPPA – semantic variant PPA, nfvPPA – nonfluent/agrammatic variant PPA.

ALLFTD Longitudinal analysis

Supplementary Fig. 23a is a Sankey diagram demonstrating the longitudinal assignment to subtype for the 58 patients who had longitudinal data and were subtypable at baseline.

Supplementary Fig. 23b and 23c shows longitudinal consistency of model staging, stratified by model subtype (b) and clinical diagnosis (c). At first follow up, 46/53 (86.8%) patients advanced (upward) to a later stage, 4/53 (7.5%) patients remained at the same stage (on the diagonal), while a further 3/53 (5.7%) patients regressed (downward) to an earlier stage, giving a longitudinal staging consistency exceeding 94.3% (50/53).

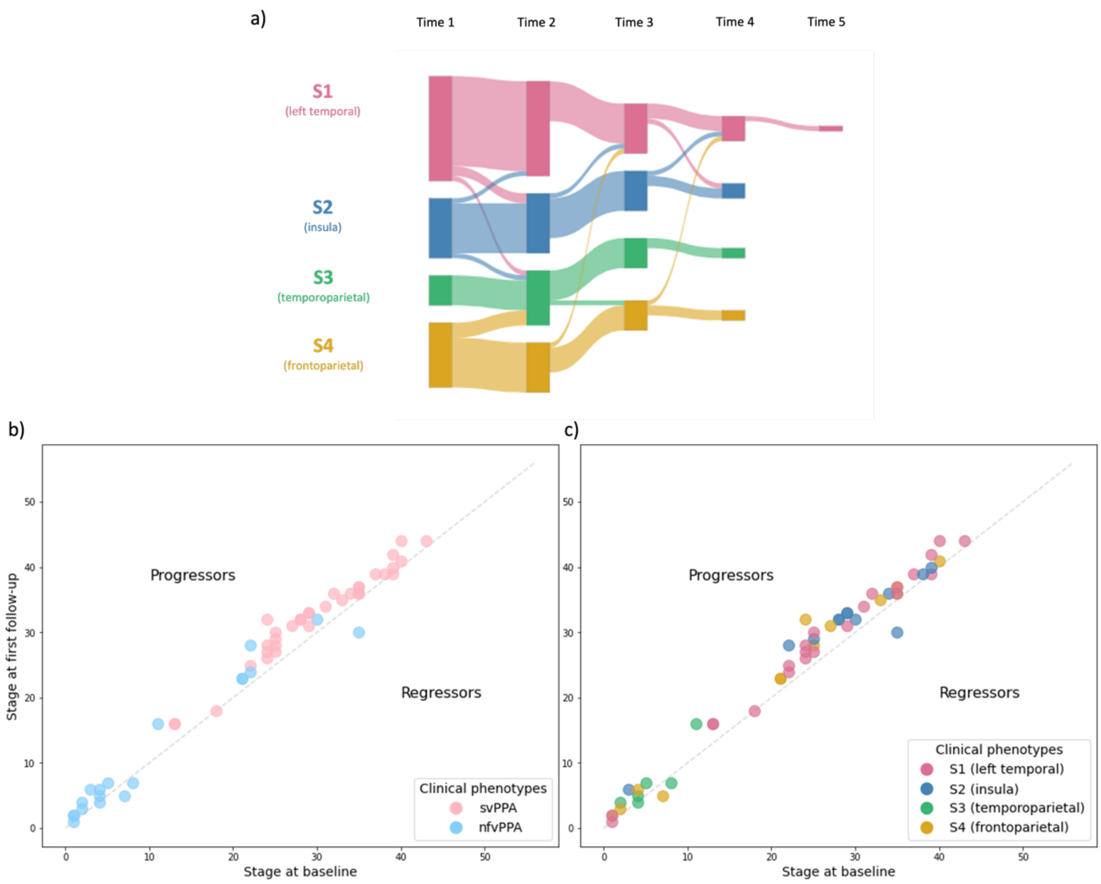

Supplementary Figure 23: a) **Sankey diagram of subtype assignment between baseline and follow-up visits in ALLFTD test set.** The bars are colour coded to represent the percentage of patients in each subtype at first follow up, stratified by their subtype assignment at baseline S1 left temporal (pink), S2 insula (blue), S3 left TPJ (green), S4 left frontal parietal (yellow). **Data-driven stage assignment at baseline and first follow up in the ALLFTD test set.** Patients are colour coded according to clinical diagnosis (b) and according to subtype assignment (c). Those above the diagonal progressed at follow up, whilst those below regressed.

Abbreviations: svPPA – semantic variant PPA, nfvPPA – nonfluent/agrammatic variant.

ALLFTD Association with neuropsychological test scores

Supplementary Fig. 24 compares the neuropsychological test scores at baseline, stratified by baseline subtype assignment. The Boston Naming Test, Digit Span Forwards and Digit Span Backwards had statistically significant ANOVAs. A full table with count, mean and standard deviation of test scores can be found for all available neuropsychological measures in Supp Table Z.

Supplementary Fig. 2 shows the comparison between baseline data driven stage and MMSE score, stratified by subtype assignment in the ALLFTD test set. Performing a multilevel linear regression, we found that the relationship (regression slope) between MMSE and baseline stage was not statistically significant in any subtype, however this is likely due to the small sample size of just n=59, with MMSE scores available for n=23/13/12/11 in S1/S2/S3/S4 respectively.

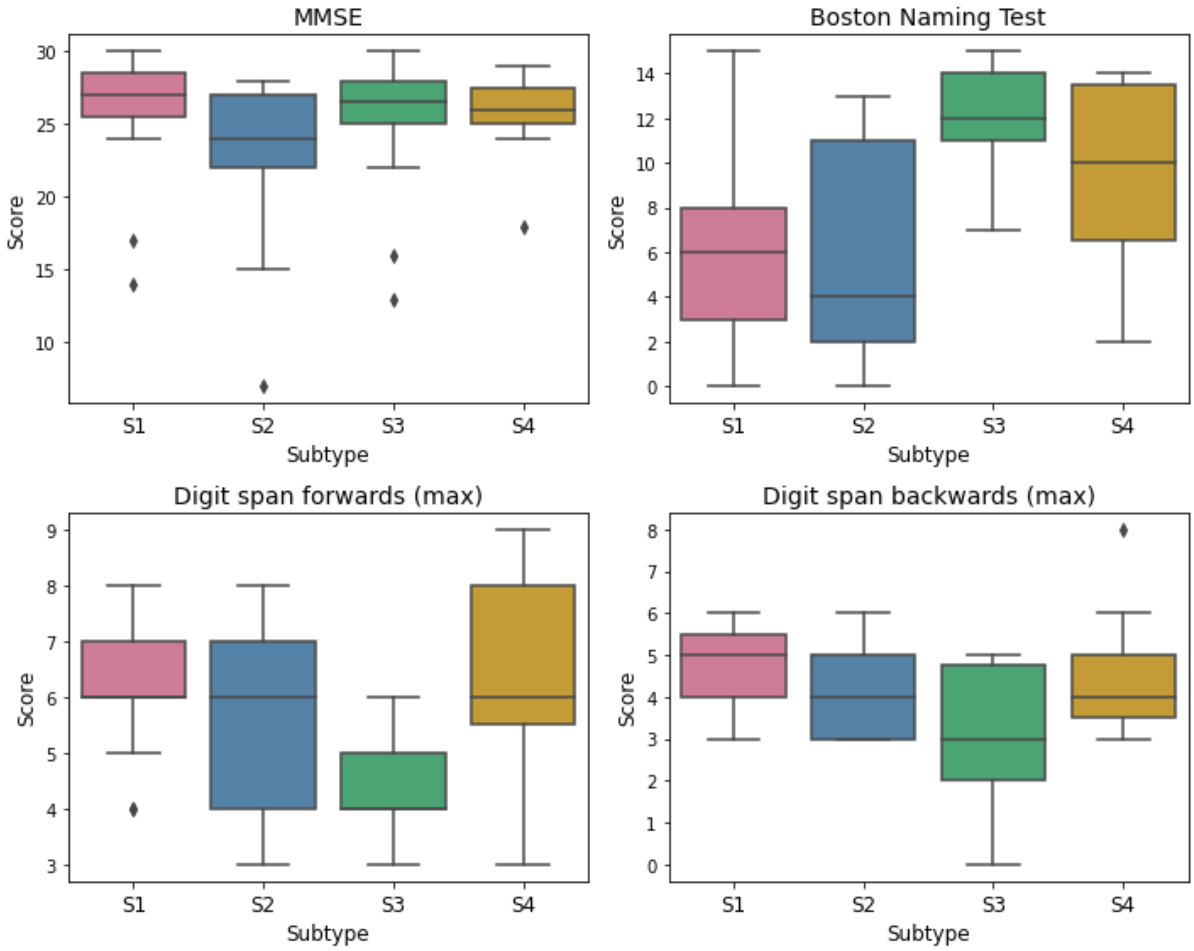

Supplementary Figure 24: **Boxplots comparing MMSE and neuropsychological scores between subtypes at baseline in ALLFTD.** The plots are colour coded by data-driven subtype – S1 left temporal (pink), S2 insula (blue), S3 temporoparietal (green), S4 frontoparietal (yellow).

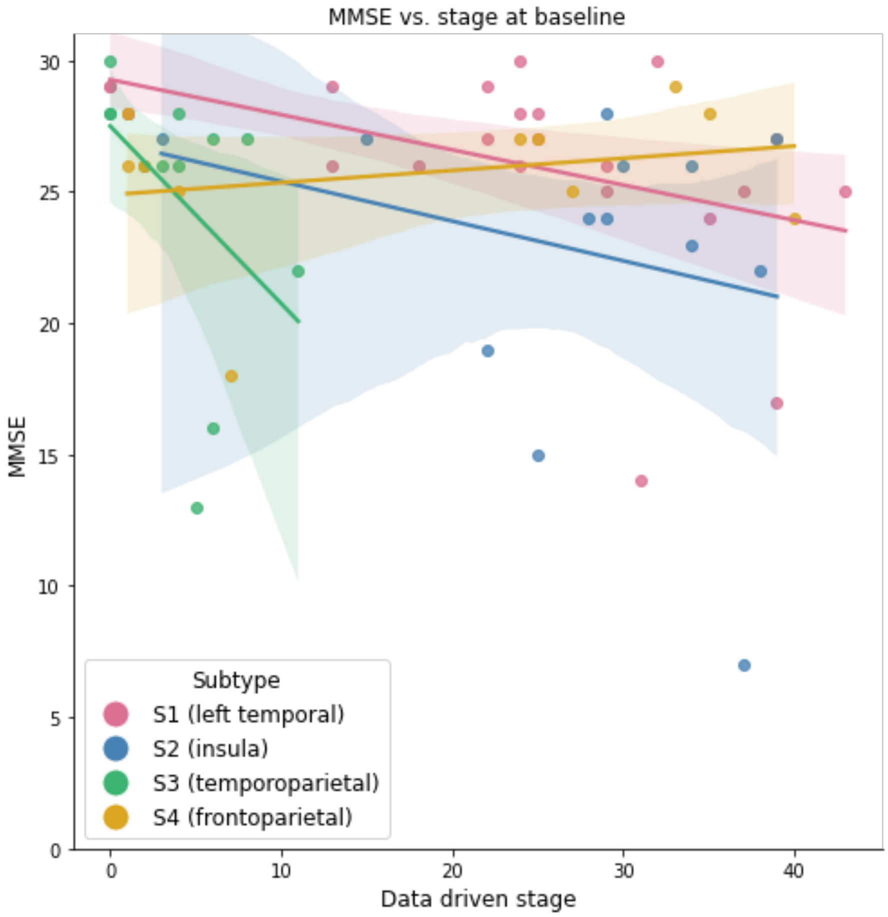

Supplementary Figure 25: **MMSE vs Stage at baseline in the ALLFTD test dataset.** The multilevel regression plots are colour coded to represent subtype assignment at baseline S1 left temporal (pink), S2 insula (blue), S3 temporoparietal (green), S4 frontoparietal (yellow).

References

1. Cardoso MJ, Modat M, Wolz R, et al. Geodesic Information Flows: Spatially-Variant Graphs and Their Application to Segmentation and Fusion. *IEEE Trans Med Imaging*. 2015;34(9):1976-1988. doi:10.1109/TMI.2015.2418298
2. Young AL, Marinescu R V., Oxtoby NP, et al. Uncovering the heterogeneity and temporal complexity of neurodegenerative diseases with Subtype and Stage Inference. *Nature Communications 2018 9:1*. 2018;9(1):1-16. doi:10.1038/s41467-018-05892-0
3. Vogel JW, Young AL, Oxtoby NP, et al. Four distinct trajectories of tau deposition identified in Alzheimer's disease. *Nat Med*. 2021;27(5):871. doi:10.1038/S41591-021-01309-6
4. Oxtoby N. Disease SNR: a feature selection framework for disease progression modelling. Published online 2023.

5. Oxtoby NP, Leyland LA, Aksman LM, et al. Sequence of clinical and neurodegeneration events in Parkinson's disease progression. *Brain*. Published online 2021:1-14. doi:10.1093/brain/awaa461
6. Fonteijn HM, Modat M, Clarkson MJ, et al. An event-based model for disease progression and its application in familial Alzheimer's disease and Huntington's disease. *Neuroimage*. 2012;60(3):1880-1889. doi:10.1016/j.neuroimage.2012.01.062
7. Henry ML, Gorno-Tempini ML. The logopenic variant of primary progressive aphasia. *Curr Opin Neurol*. 2010;23(6):633. doi:10.1097/WCO.0B013E32833FB93E
8. Gorno-Tempini ML, Hillis AE, Weintraub S, et al. Classification of primary progressive aphasia and its variants. *Neurology*. 2011;76(11):1006.
